# [^99m^Tc]Tc-PentixaTec: development, extensive pre-clinical evaluation, and first human experience

**DOI:** 10.1007/s00259-023-06395-x

**Published:** 2023-08-19

**Authors:** Matthias Konrad, Andreas Rinscheid, Georgine Wienand, Bernd Nittbaur, Hans-Jürgen Wester, Tilman Janzen, Constantin Lapa, Christian Helmut Pfob, Margret Schottelius

**Affiliations:** 1 Chair for Pharmaceutical Radiochemistry, Faculties of Chemistry and Medicine, Technische Universität München, 85748 Garching, Germany; 2 Medical Physics and Radiation Protection, University Hospital Augsburg, Stenglinstrasse 2, 86156 Augsburg, Germany; 3 Nuclear Medicine, Faculty of Medicine, University of Augsburg, Stenglinstrasse 2, 86156 Augsburg, Germany; 4 Translational Radiopharmaceutical Sciences, Department of Nuclear Medicine and Department of Oncology, Centre Hospitalier Universitaire Vaudois (CHUV) and University of Lausanne (UNIL), Rue du Bugnon 25A, Agora, CH-1011 Lausanne, Switzerland; 5 AGORA, Pôle de Recherche Sur Le Cancer, 1011 Lausanne, Switzerland; 6 SCCL Swiss Cancer Center Leman, 1011 Lausanne, Switzerland

**Keywords:** CXCR4 imaging, Cancer, SPECT/CT, Planar imaging, [^99m^Tc]Tc-PentixaTec, Pentapeptide tracer

## Abstract

**Purpose:**

The clinical success non-invasive imaging of CXCR4 expression using [^68^ Ga]Ga-PentixaFor-PET warrants an expansion of the targeting concept towards conventional scintigraphy/SPECT with their lower cost and general availability. To this aim, we developed and comparatively evaluated a series of ^99m^Tc-labeled cyclic pentapeptides based on the PentixaFor scaffold.

**Methods:**

Six mas_3_-conjugated CPCR4 analogs with different 4-aminobenzoic acid (Abz)-D-Ala-D-Arg-aa_3_ linkers (**L1–L6**) as well as the corresponding HYNIC- and N_4_-analogs of **L6-**CPCR4 were synthesized via standard SPPS. Competitive binding studies (IC_50_ and IC_50_inv) were carried out using Jurkat T cell lymphoma cells and [^125^I]FC-131 as radioligand. Internalization kinetics were investigated using hCXCR4-overexpressing Chem-1 cells. Biodistribution studies and small animal SPECT/CT imaging (1 h p.i.) were carried out using Jurkat xenograft bearing CB17/SCID mice. Based on the preclinical results, [^99m^Tc]Tc-N_4_-**L6-**CPCR4 ([^99m^Tc]Tc-PentixaTec) was selected for an early translation to the human setting. Five patients with hematologic malignancies underwent [^99m^Tc]Tc-N_4_-**L6**-CPCR4 SPECT/planar imaging with individual dosimetry.

**Results:**

Of the six mas_3_-conjugated peptides, mas_3_**-L6**-CPCR4 (mas_3_-dap-r-a-Abz-CPCR4) showed the highest CXCR4 affinity (IC_50_ = 5.0 ± 1.3 nM). Conjugation with N_4_ (N_4_-**L6**-CPCR4) further improved hCXCR4 affinity to 0.6 ± 0.1 nM. [^99m^Tc]Tc-N_4_-**L6-**CPCR4 also showed the most efficient internalization (97% of total cellular activity at 2 h) and the highest tumor accumulation (8.6 ± 1.3% iD/g, 1 h p.i.) of the compounds investigated. Therefore, [^99m^Tc]Tc-N_4_-**L6**-CPCR4 (termed [^99m^Tc]Tc-PentixaTec) was selected for first-in-human application. [^99m^Tc]Tc-PentixaTec was well tolerated, exhibits a favorable biodistribution and dosimetry profile (2.1–3.4 mSv per 500 MBq) and excellent tumor/background ratios in SPECT and planar imaging.

**Conclusion:**

The successive optimization of the amino acid composition of the linker structure and the N-terminal ^99m^Tc-labeling strategies (mas_3_ vs HYNIC vs N_4_) has provided [^99m^Tc]Tc-PentixaTec as a novel, highly promising CXCR4-targeted SPECT agent for clinical application. With its excellent CXCR4 affinity, efficient internalization, high uptake in CXCR4-expressing tissues, suitable clearance/biodistribution characteristics, and favorable human dosimetry, it holds great potential for further clinical use.

**Graphical abstract:**

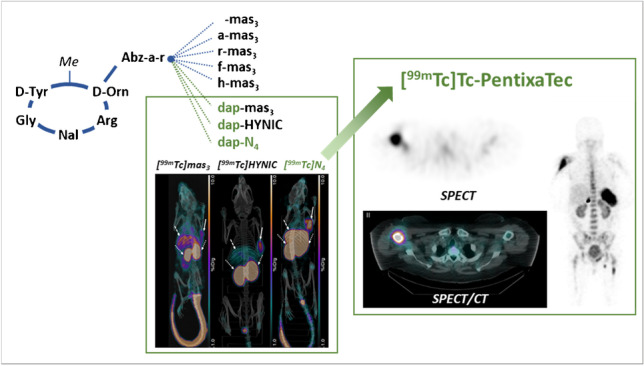

**Supplementary Information:**

The online version contains supplementary material available at 10.1007/s00259-023-06395-x.

## Introduction

The C-X-C motif chemokine receptor 4 (CXCR4) is a key player in tumor growth and the process of metastasis and as such is a highly attractive target in nuclear oncology. A theranostic approach (mainly for hematologic malignancies) based on the complementary cyclic pentapeptides [^68^ Ga]Ga-PentixaFor and [^177^Lu/^90^Y]Lu/Y-PentixaTher has therefore been successfully implemented into clinical practice [1–8]. As CXCR4 is also endogenously expressed on various leukocyte subtypes [9], diverse applications in inflammatory and/or infectious conditions such as acute myocardial infarction, atherosclerosis, stroke, urinary tract, and chronic bone infections have been described [10–15].

In all of these indications, [^68^ Ga]Ga-Pentixafor was shown to allow for sensitive and high-contrast positron emission tomography (PET). To date, it is the only CXCR4-targeted imaging agent that has achieved broad clinical applicability so far [16–18]. However, given the continuously expanding field of clinical indications for non-invasive in vivo CXCR4 visualization and quantification, development of a ligand amenable to radiolabeling with gamma emitters (such as ^99m^Tc) for use in conventional scintigraphy or single photon emission computed tomography (SPECT) with its lower costs and general availability would be highly desirable.

This need has been recognized and has fueled the recent development and preclinical characterization of ^99m^Tc-labeled CXCR4-targeted tracers [19–22]. Of these, only one compound has been translated towards human application so far, namely [^99m^Tc]Tc-CXCR4-L (= [^99m^Tc]HYNIC-CPCR4 = *cyclo*(D-Tyr-*N*-Me-D-Orn([^99m^Tc]Tc-HYNIC)-Arg-2-Nal-G)) [21, 22], which is based on the same pentapeptide backbone as PentixaFor. It displayed rapid whole-body clearance in healthy volunteers, albeit with significant bowel accumulation at time points > 2 h p.i., and allowed for successful visualization of glioma lesions in patients [22].

Our own experience with PentixaFor-based radioligands, however, has consistently demonstrated that even minor modifications of the peptide scaffold (either directly at the D-Orn side chain or at the far end of the D-Orn-AMBS-moiety) may lead to substantial losses in CXCR4 binding affinity [23–25]. Thus, implementation of a Tc-labeling strategy based on the PentixaFor-backbone represents a challenge, since the required structural modifications to accommodate ^99m^Tc-chelation are usually quite extensive [26]. In a previous study on PentixaFor- and PentixaTher-based DOTA-conjugated tracers, replacement of the AMBS- by a Abz-a-r-linker had emerged as a powerful tool to improve ligand affinity and internalization. Furthermore, the flexibility of the tracer scaffold towards structural modifications at the far end of the linker was substantially improved [24]. We therefore selected the CPCR4-Abz-a-r scaffold as a starting point for the development of novel ^99m^Tc-labeled analogs with further optimized linkers. Upon selection of the most promising peptide-linker construct using mas_3_-based labeling chemistry, other ^99m^Tc-labeling methods (HYNIC, N_4_) were also implemented, and a comparative in-depth preclinical characterization was performed. Based on the particularly promising in vitro and in vivo characteristics of [^99m^Tc]Tc-N_4_-dap-r-a-Abz-CPCR4 ([^99m^Tc]Tc-N_4_-**L6**-CPCR4, [^99m^Tc]Tc-PentixaTec), we also report its first in human application in four patients with hematological malignancies.

## Materials and methods

### Precursor synthesis

All compounds in this study were synthesized by combining solid phase peptide synthesis (SPPS) using a standard Fmoc-protocol and solution phase fragment condensation strategies. Briefly, the pentapeptide backbone CPCR4 was synthesized according to a literature protocol [27] and then functionalized with the corresponding linkers (**L1–L6**) and respective Tc-chelators mas_3_, HYNIC and N_4_ as described in detail in the supplementary information.

### Radiolabeling

Labeling with ^99m^Tc was carried out using kit-like lyophilized reaction vials (see supplementary material), based on established labeling protocols for mas_3_- [28], HYNIC- [29], and N_4_-conjugated peptide tracers [30] with minor modifications. Details are provided in the supplementary material.

### Determination of lipophilicity (log*D*)

The lipophilicity (log*D*, partition coefficient between n-octanol and PBS, pH 7.4) of all ^99m^Tc-labeled compounds in this study was determined using a modified shake-flask method as described [31].

### In vitro stability assay

One hundred microliters of [^99m^Tc]Tc-PentixaTec was added to 1 mL of saline (0.9% NaCl), 1 mL serum originating from 2.7 mL whole blood with 1.6 mg EDTA/mL, 3.2% citrate whole blood (citrate concentration 0.106 M), 0.1 mg/mL DTPA in saline (0.9% NaCl), 10% sodium ascorbate in water, 5% human serum albumin (HSA; Humanalbin®, CSL Behring, Marburg, Germany), 2.5% HSA (1:1 dilution of Humanalbin® solution with 0.9% NaCl), and NH_4_-Heparin 16I.E./mL whole blood, respectively. Solutions were incubated for up to 13 h at room temperature. Sequential radio-thin layer chromatography was performed after 20 min, 40 min, 100 min, 2.6 h, 3.6 h, 4.4 h, and 13 h of incubation, and the amount of intact ligand was analyzed by integration of the TLC chromatogram.

### In vitro evaluation

#### Determination of affinity to hCXCR4 and mCXCR4 (IC_50_)

To determine ligand affinity (IC_50_) to human CXCR4 (hCXCR4), competition binding experiments using Jurkat cells and [^125^I]FC-131 as standard radioligand were carried out as previously described [32]. Ligand affinity to mouse CXCR4 (mCXCR4) was determined using the same experimental protocol, but HEK cells stably expressing mCXCR4 and [^125^I]CPCR4.3 [33] as radioligand.

#### Determination of inv-IC_50_ (hCXCR4)

For the determination of the “inverse IC_50_” (inv-IC_50_) of the ^99m^Tc-labeled compounds in this study, the same experimental protocol as used for the IC_50_-determination was employed. However, the respective ^99m^Tc-labeled peptide of interest was used as radioligand (0.2 nM), and unlabeled FC-131 was used as a standard competitor. Both IC_50_ values and inv-IC_50_ values were calculated using GraphPad Prism 6 (GraphPad Software Inc., San Diego, USA).

#### Dual tracer internalization studies

Dual tracer internalization studies using [^125^I]FC-131 as internal reference were conducted in hCXCR4 expressing Chem-1 cells in analogy to a previously established protocol [34]. A detailed assay protocol is provided in the supplementary material section.

### In vivo evaluation

All animal experiments were conducted in accordance with general animal welfare regulations in Germany and the institutional guidelines for the care and use of animals.

#### Biodistribution studies

Female CB17/SCID mice bearing subcutaneous Jurkat human T cell lymphoma xenografts (for more detailed information, see supplemental material) were injected intravenously with the respective ^99m^Tc-labeled tracer (5–20 MBq, 0.1–0.2 nmol/mouse) under isofluorane anesthesia and were sacrificed at 1 h post-injection (p.i.). The organs of interest were dissected, and the activity concentration in weighed tissue samples was quantified using a WIZARD^2^® 2480 automatic γ-Counter from Perkin Elmer (Waltham MA, USA). Biodistribution data are given in %iD/g and represent means ± SD (groups of *n* = 4–5 animals). Statistical analysis (*t*-test) was performed using GraphPad Prism.

#### Small animal SPECT/CT imaging

SPECT/CT imaging was performed on a VECTor^4^ small-animal SPECT/PET/OI/CT scanner (MILabs BV, Utrecht, The Netherlands). Static images were acquired with 45-min acquisition time using the HE-GP-RM collimator and a stepwise multi-planar bed movement. All images were reconstructed using the MILabs Rec software (version 10.02) and a pixel-based Similarity-Regulated Ordered Subsets Expectation Maximization (SROSEM) algorithm with a window-based scatter correction (20% below and 20% above the photopeak, respectively; voxel size CT: 80 µm, voxel size SPECT: 0.8 mm, 1.6 mm (FWHM) Gaussian blurring post-processing filter, with calibration factor in kBq/mL and decay correction, no attenuation correction). Image analysis was carried out using PMOD 4.0 (PMOD TECHNOLOGIES LLC, Fällanden, Switzerland).

### Patient studies

#### Patients

The use of [^99m^Tc]Tc-PentixaTec was in compliance with the German Medicinal Products Act, AMG §13 No 2b and reported to the regulatory office (Regierung von Oberbayern). The analysis of patient data was approved by the Ethics Committee of Ludwig-Maximilians Universität München (permit 22–0850).

Five patients (age, 48–72 y, median 66 y) with a history of hematological malignancy underwent imaging after intravenous injection of a median of 558 MBq (range, 417–601) [^99m^Tc]Tc-PentixaTec. One patient suffered from marginal zone lymphoma, one from B-NHL, and the remaining three individuals had a history of multiple myeloma. Safety was assessed by monitoring adverse events after administration of [^99m^Tc]PentixaTec.

#### Human [^99m^Tc]Tc-PentixaTec gamma camera imaging

[^99m^Tc]Tc-PentixaTec scans were performed using a dual head gamma camera (GE Discovery NM/CT 670 Pro, Milwaukee, WI, USA). In the four patients undergoing dosimetry, planar dynamic whole body scans were performed 5 min, 30 min, and 1 h with a bed speed of 30 cm/min; 2 h, 3 h, and 5 h with a speed of 12 cm/min; and 24 h with a speed of 5 cm/min after tracer injection. SPECT/CT imaging was performed following the 3-h planar imaging with 60 views and a frame duration of 8 s. Attenuation maps were generated on the basis of low-dose CT.

#### Blood clearance

Venous blood samples (2.7 mL) were drawn directly before each planar scan. A cross-calibrated well-counter (Digibase, Ametek, Oak Ridge, TN, USA) was used to measure activity in the blood samples, resulting in the average activity per mL blood. Plasma half-life was calculated using NUKFIT software [35].

#### Radiation dosimetry and biodistribution

Radiation dosimetry calculations were performed using the RADAR (Radiation Dose Assessment Resource) [36] method as implemented in the OLINDA/EXM software and according to the recommendations of the Medical Internal Radiation Dose (MIRD) committee [37]. The individual absorbed organ doses (ODs) and the effective doses (EDs) were corrected to consider the current tissue weighting factors of ICRP 103 [38]. For a detailed description of image analysis for dosimetry, please refer to the supplementary materials section.

## Results and discussion

### Identification of a suitable linker unit

In a first step, the effect of direct mas_3_-functionalization of the r-a-Abz-CPCR4 backbone on CXCR4 affinity was investigated. Compared to the corresponding ^nat^Ga/^nat^Lu-complexes of DOTA-r-a-Abz-CPCR4, which had shown CXCR4 affinities of 0.4 ± 0.1 and 1.5 ± 0.1 nM, respectively [24], the affinity of mas_3_-r-a-Abz-CPCR4 (mas_3_-**L1-**CPCR4), despite being in the same range as the clinically used reference ligands ^nat^Ga-PentixaFor and ^nat^Lu-PentixaTher (Table 1), was substantially lower. To investigate if the interaction with the CXCR4 binding pocket may be improved by a linker extension, additional neutral (D-Ala = a), aromatic (D-Phe = f), or cationic (D-Arg = r, D-His = h, D-Dap = dap) amino acids were introduced (Table 1). Of all modifications, only small cationic side chains (h and dap in **L5** and **L6**, respectively) were tolerated without compromising CXCR4-affinity. The fourfold increased affinity of mas_3_-**L6-**CPCR4 compared to mas_3_-**L1-**CPCR4 prompted its further preclinical evaluation. Furthermore, the **L6-**CPCR4 construct was chosen for further functionalization with HYNIC and N_4_, allowing a side-by-side comparison of alternative ^99m^Tc-labeling strategies.

**Table 1:** CXCR4 affinities of the novel mas_3_-conjugated ligands compared to selected reference compounds

Ligand		IC_50_ (nM)
^nat^Ga-PentixaFor		24.9 ± 2.5
^nat^Lu-PentixaTher		14.6 ± 1.0
mas_3_-r-a-Abz-CPCR4	(mas_3_-**L1-**CPCR4)	20.6 ± 7.5
mas_3_-**a**-r-a-Abz-CPCR4	(mas_3_-**L2-**CPCR4)	32.4 ± 9.9
mas_3_-**r**-r-a-Abz-CPCR4	(mas_3_-**L3-**CPCR4)	184 ± 25
mas_3_-**f**-r-a-Abz-CPCR4	(mas_3_-**L4-**CPCR4)	3490 ± 502
mas_3_-**h**-r-a-Abz-CPCR4	(mas_3_-**L5-**CPCR4)	11.0 ± 1.3
mas_3_-**dap**-r-a-Abz-CPCR4	(mas_3_-**L6-**CPCR4)	5.0 ± 1.3

### Synthesis of L6-CPCR4-based chelator conjugates

Synthetically, all peptides (Tables 1 and 2) were accessible via a combined solid phase peptide synthesis/solution phase fragment condensation strategy (for details, see supplementary material). Since the conjugation of the complete Abz-a-r-X-mas_3_ constructs to the CPCR4 backbone (as employed for peptides mas_3_-**L3**/**L4**/**L5**-CPCR4) was found to be inefficient, two successive coupling steps were employed for the synthesis of all other peptides. For the peptides mas_3_/HYNIC/N_4_-**L6-**CPCR4, this involved attachment of the Fmoc-protected Abz-a-r(Pbf)-dap(Boc)-linker (**L6**) to the peptide core as a first step, and, after Fmoc deprotection and purification of the CPCR4-**L6**-scaffold, functionalization with the corresponding technetium-chelator, followed by final cleavage of all acid-labile protecting groups. In the case of HYNIC-**L6-**CPCR4, the formation of trifluoroacetyl-HYNIC as a side product during the last deprotection step limited the yield of the isolated product to 3.4% based on **L6**-CPCR4. During the synthesis of N_4_-**L6-**CPCR4, pre-activation of (Boc)_4_N_4_-COOH with HOAt/HATU and the use of 2,4,6-collidine as a base was found to successfully prevent the previously observed elimination of Boc-protected 1,2- ethylenediamine from the (Boc)_4_N_4_ moiety during the condensation reaction. N_4_-**L6-**CPCR4 was thus obtained in reasonable yields of 12–17% based on **L6-**CPCR4 after RP-HPLC purification.

**Table 2:** CXCR4 affinities, internalization (in % of the reference ([^125^I]FC-131)), and lipophilicities (log*D*) of the novel ligands and selected reference compounds

Ligand	IC_50_ (nM)	Tracer	inv-IC_50_ (nM)	Internalization (% of reference)*	Lipophilicity ( log*D*)
^nat^Ga-PentixaFor	24.9 ± 2.5	[^68^ Ga]Ga-PentixaFor	-	-	− 2.90
^nat^Lu-PentixaTher	14.6 ± 1.0	[^177^Lu]Lu-PentixaTher	1.9 ± 1.1	283 (120 min)	− 1.76
HYNIC-CPCR4^#^	-	[^99m^Tc]Tc-HYNIC-CPCR4^#^	< 0.1	29 (90 min)	− 1.84
mas_3_-**L6-**CPCR4	5.0 ± 1.3	[^99m^Tc]Tc-mas_3_-**L6-**CPCR4	3.7 ± 0.8	525 (90 min)	− 1.54
HYNIC-**L6**-CPCR4	4.1 ± 1.5	[^99m^Tc]Tc-HYNIC-**L6**-CPCR4	4.2 ± 1.5	503 (90 min)	− 2.74
N_4_-**L6**-CPCR4 (PentixaTec)	0.60 ± 0.06	[^99m^Tc]Tc-N_4_-**L6**-CPCR4 ([^99m^Tc]Tc-PentixaTec)	10.2 ± 2.4	694 (120 min)	− 1.75

*Specific internalization (total internalized activity corrected by internalization in the presence of 10 µM AMD3100) of the reference compound [^125^I]FC-131 was determined in the respective dual tracer experiment and used for data normalization

^**#**^Name in the literature [21, 22]: CXCR4-L

### Radiolabeling

Both [^99m^Tc]Tc-mas_3_-**L6**-CPCR4 and [^99m^Tc]Tc-N_4_-**L6**-CPCR4 were consistently obtained in > 95% radiochemical yield and radiochemical purity, respectively, using prefabricated, lyophilized radiolabeling kits. For in vitro and in vivo applications, the radiolabeling products were thus only diluted with saline or PBS and sterile filtered without the requirement for further purification. In contrast, ^99m^Tc-labeling yields for [^99m^Tc]Tc-HYNIC-**L6**-CPCR4 remained below 50% (*n* = 7), necessitating cartridge purification before further use. Interestingly, radiolabeling yields for the HYNIC-containing reference compound [^99m^Tc]Tc-HYNIC-CPCR4 ([^99m^Tc]Tc-CXCR4-L [21, 22]) were always > 85%, suggesting a potential influence of the adjacent amino acid side chains of the linker unit in [^99m^Tc]Tc-HYNIC-**L6**-CPCR4 on the labeling efficiency. Cation-pi interactions between the cationic D-Dap- and/or D-Arg-side chain and the aromatic system in HYNIC may lead to a decreased electron donating capacity of the HYNIC moiety and thus contribute to the observed reduction in ^99m^Tc-labeling efficiency [39].

### In vitro characterization

The in vitro characteristics of [^99m^Tc]mas_3_-**L6**-CPCR4, [^99m^Tc]HYNIC-**L6**-CPCR4, and [^99m^Tc]N_4_-**L6**-CPCR4 in comparison to selected reference compounds [1, 22, 32] are summarized in Table 2.

As their mas_3_-counterpart, unlabeled HYNIC-**L6**-CPCR4 and N_4_-**L6**-CPCR4 showed excellent and even improved hCXCR4 affinities, with an almost ninefold increase in affinity observed for the N_4_-conjugated analog, highlighting the suitability of the **L6**-linker for accommodating various N-terminal modifications without challenging receptor affinity. Chelation of ^99m^Tc(V), however, is known to induce substantial structural changes at the chelator site, as determined by the geometry and charge of the resulting ^99m^TcO-mas_3_-, ^99m^Tc-HYNIC/EDDA, and ^99m^TcO_2_-N_4_-complexes [26, 40]. Determining their influence on the CXCR4-affinity of the corresponding tracers is thus indispensable. However, there is no stable technetium isotope, allowing the preparation of cold reference compounds, and the synthesis of the corresponding chemically equivalent Re-complexes requires harsh conditions [41], long reaction times [42], and substantial precursor amounts. For this reason, we directly determined the CXCR4-affinity of the respective ^99m^Tc-labeled peptides (and of [^177^Lu]Lu-PentixaTher as reference) using an inverse IC_50_ setup (inv-IC_50_). Here, the competition binding study is performed using a fixed concentration of the respective radioligand of interest (0.2 nM) and FC-131 as standard competitor. Under these conditions, a higher absolute inv-IC_50_ corresponds to a higher CXCR4 affinity of the radioligand, since more unlabeled FC-131 is needed to replace receptor-bound tracer.

In this setting, both [^99m^Tc]Tc-mas_3_-**L6**-CPCR4 and [^99m^Tc]Tc-HYNIC-**L6**-CPCR4 showed a twofold increase in CXCR4 affinity compared to [^177^Lu]Lu-PentixaTher (*p* = 0.001 and 0.002, respectively).

Surprisingly, [^99m^Tc]Tc-HYNIC-CPCR4 (= [^99m^Tc]Tc-CXCR4-L), the only clinically used ^99m^Tc-labeled CXCR4 ligand reported so far [21, 22], was displaced by FC-131 even at concentrations below 0.1 nM, showing virtually no CXCR4-affinity in this assay, which is in contradiction to its reported K_D_ of 3.3 nM (in Du-4475 breast cancer cells) [21]. Again, the compound with the highest inv-IC_50_ value in this study and thus the highest CXCR4-affinity was [^99m^Tc]Tc-N_4_-**L6**-CPCR4, with a fivefold higher affinity than [^177^Lu]Lu-PentixaTher (Table 2, *p* < 0.0001).

**Fig. 1:**
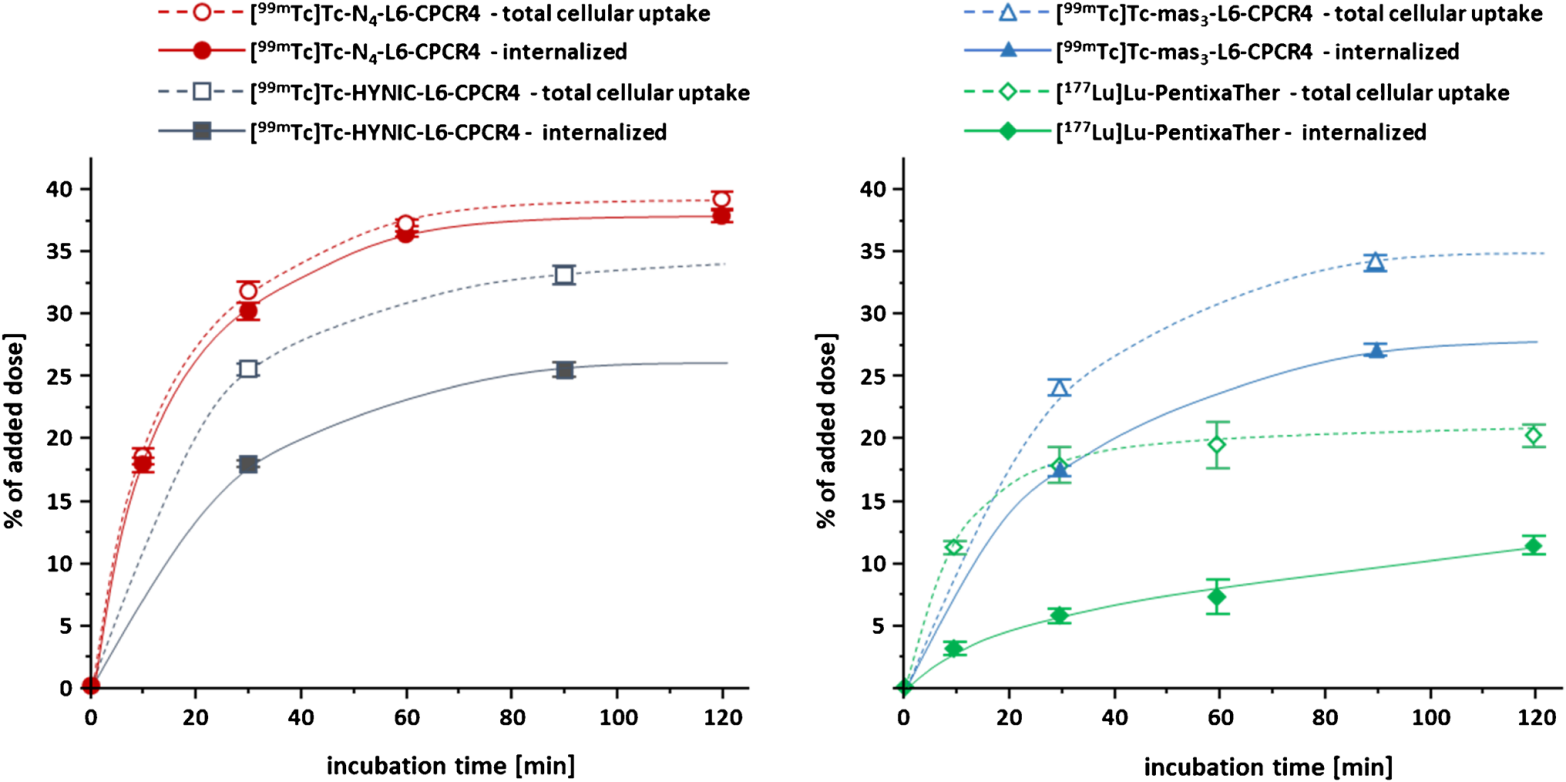
Internalization kinetics of [^99m^Tc]Tc-HYNIC-**L6**-CPCR4, [^99m^Tc]Tc-N_4_-**L6**-CPCR4 (left panel), [^99m^Tc]Tc-mas_3_-**L6**-CPCR4, and [^177^Lu]Lu-PentixaTher (right panel). Solid lines indicate internalized activity, and dashed lines indicate total cellular activity [% added dose]. Experiments were performed at 37° at a ligand concentration of 0.2 nM. Data are means ± SD of *n* = 3 independent experiments

As shown in Fig. 1, [^99m^Tc]Tc-N_4_-**L6**-CPCR4 also shows the highest internalization of all compounds investigated, both with regard to absolute intracellular tracer accumulation (app. 40% of added dose at 2 h, area under the curve (AUC) = 3799 vs 2430, 2370, and 860 for the mas_3_- and HYNIC analogs and [^177^Lu]Pentixather, respectively) and to the ratio of internalized vs total cellular activity (app. 97% of total cellular activity internalized). Generally, all three Abz-a-r-linker containing peptides show particularly high internalization (absolute ([% of added dose], AUC), in % of total cellular uptake (Fig. 1) and in % of the internal reference [^125^I]FC-131 (Table 2)). This effect seems to be primarily caused by the Abz-based linker, which induces a shift from an antagonistic (as in, e.g., [^177^Lu]Lu-PentixaTher) towards a (partial) agonistic profile of the tracers, as shown in a previous study on the corresponding DOTA-r-a-Abz-conjugated CXCR4 ligands [24]. However, compared to these compounds, the additional dap-residue in **L6** seems to further promote internalization efficiency, as exemplified by 80 and 77% internalization (in % of total activity at 90 min, Fig. 1) for [^99m^Tc]Tc-mas_3_-**L6**-CPCR4 and [^99m^Tc]Tc-HYNIC-**L6**-CPCR4 vs 65% for [^177^Lu]Lu-DOTA-r-a-Abz-CPCR4 [24].

### In vivo biodistribution and small animal SPECT/CT imaging

Subsequently, the in vivo biodistribution profiles of [^99m^Tc]Tc-mas_3_-**L6**-CPCR4, [^99m^Tc]Tc-HYNIC-**L6**-CPCR4, and [^99m^Tc]Tc-N_4_-**L6**-CPCR4 were investigated in a comparative in vivo biodistribution study (1 h p.i.) in Jurkat human T cell lymphoma xenograft-bearing mice (Table 3).

**Table 3:** Biodistribution of [^99m^Tc]Tc-mas_3_-L6-CPCR4, [^99m^Tc]Tc-HYNIC-L6-CPCR4, and [^99m^Tc]Tc-N_4_-L6-CPCR4 in Jurkat xenograft-bearing female CB17 SCID mice (*n* = 5) at 1 h p.i. Data are given in %iD/g and are means ± SD

Organ	[^99m^Tc]Tc-mas_3_-L6-CPCR4	[^99m^Tc]Tc-HYNIC-L6-CPCR4	[^99m^Tc]Tc-N_4_-L6-CPCR4 ([^99m^Tc]Tc-PentixaTec)
Blood	2.3 ± 0.5	1.6 ± 0.6	1.6 ± 0.2
Heart	1.8 ± 0.4	0.8 ± 0.3	1.2 ± 0.1
Lung	6.0 ± 1.1	1.9 ± 0.4	4.0 ± 0.6
Liver	8.4 ± 1.5	2.6 ± 0.2	7.7 ± 0.7
Pancreas	0.8 ± 0.2	0.4 ± 0.3	0.6 ± 0.1
Spleen	3.8 ± 0.4	2.4 ± 1.1	5.6 ± 0.8
Stomach	2.4 ± 0.4	2.0 ± 0.7	2.0 ± 0.4
Intestines	1.6 ± 0.2	0.6 ± 0.1	0.9 ± 0.1
Kidneys	22.8 ± 5.6	29.5 ± 4.4	37.4 ± 2.7
Muscle	0.5 ± 0.1	1.5 ± 1.1	0.3 ± 0.05
Femur	1.6 ± 0.6	0.8 ± 0.4	1.3 ± 0.3
Tumor	6.6 ± 1.0	8.0 ± 2.2	8.6 ± 1.3

**Fig. 2:**
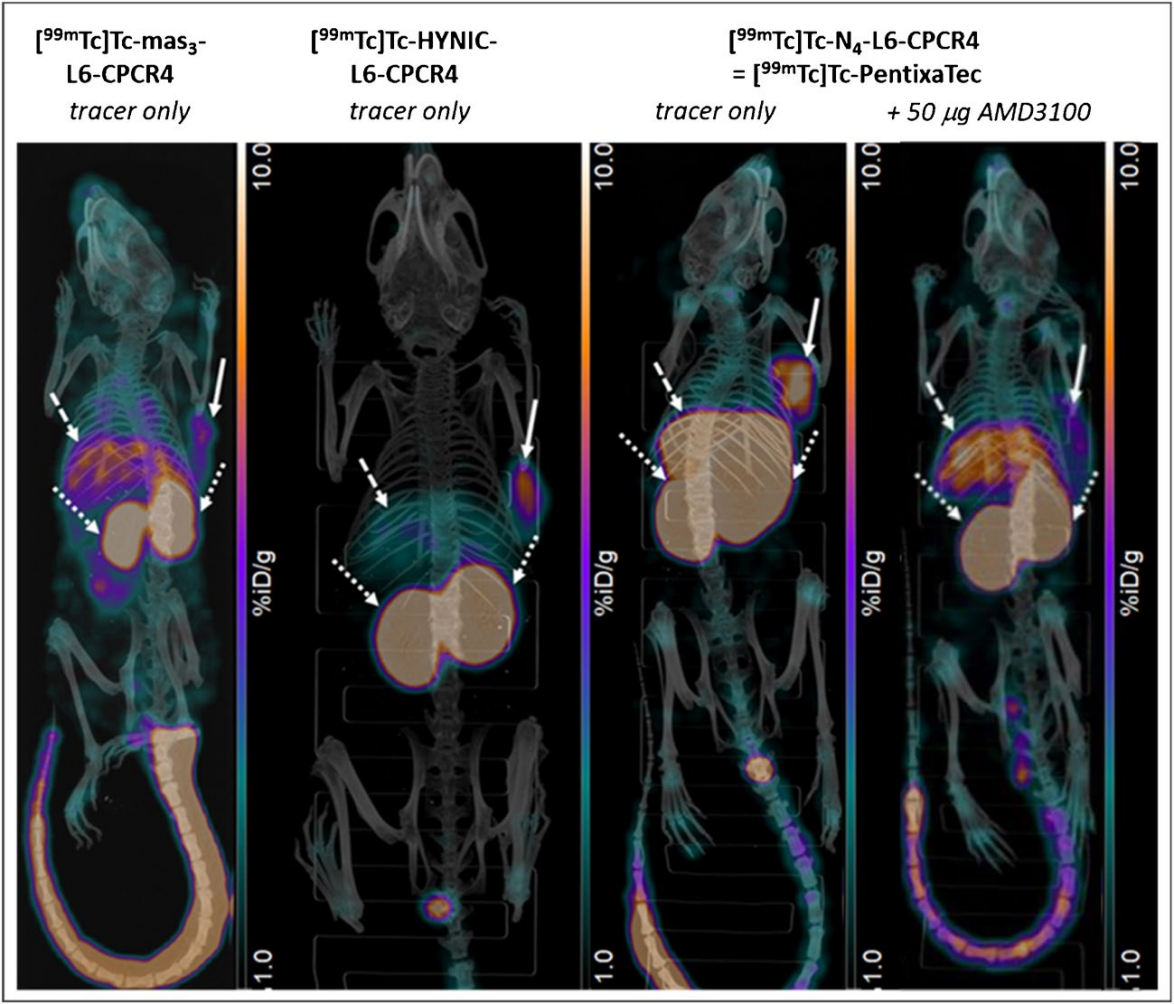
Maximum-intensity projection images obtained from static SPECT/CT imaging of Jurkat xenograft-bearing CB17 SCID mice at 1 h p.i.; solid arrows indicate tumor, dashed arrows indicate the liver, and pointed arrows indicated the kidneys

In accordance with its pronounced hydrophilicity (Table 2), [^99m^Tc]Tc-HYNIC-**L6**-CPCR4 showed the most rapid overall background clearance, with the liver, the kidneys, and the tumor being the only organs with an activity accumulation above background level (*p* < 0.0001). This is also reflected by the excellent imaging contrast observed in small animal [^99m^Tc]Tc-HYNIC-**L6**-CPCR4 SPECT/CT in a Jurkat tumor bearing mouse (Fig. 2). In contrast, for the most lipophilic compound in this series, [^99m^Tc]Tc-mas_3_-**L6**-CPCR4, with its borderline logD of − 1.54, increased background activity levels in all organs were observed (Table 3, Fig. 2). Although most of the tracer is renally excreted, the almost threefold higher intestinal activity uptake of [^99m^Tc]Tc-mas_3_-**L6**-CPCR4 compared to [^99m^Tc]Tc-HYNIC-**L6**-CPCR4 (*p* < 0.0001) and [^99m^Tc]Tc-N_4_-**L6**-CPCR4 (*p* < 0.0001) hints towards a certain degree of hepatobiliary clearance, which generally is deemed an unfavorable feature for high-contrast imaging, especially at later time points. Of the compounds investigated, [^99m^Tc]Tc-mas_3_-**L6**-CPCR4 shows the lowest tumor uptake (Table 3, Fig. 2), which is in line with its lower CXCR4 affinity compared to the other two tracers in this study.

In accordance with its particularly high CXCR4 affinity and internalization efficiency, [^99m^Tc]Tc-N_4_-**L6**-CPCR4 shows enhanced tumor accumulation compared to its mas_3_- and HYNIC-analogs. However, this difference is not as marked as anticipated (*p* = 0.002 and *p* = 0.5, respectively). Conversely, all tissues known to be mCXCR4-positive, i.e., lung, liver, and spleen [43], show notable [^99m^Tc]N_4_-**L6**-CPCR4 uptake. We had previously encountered the same effect for [^177^Lu]Lu-DOTA-r-a-Abz-*iodo*CPCR4 in direct comparison with [^177^Lu]Lu-PentixaTher at 1 h p.i. [24]. The unexpectedly low tumor accumulation of the [^177^Lu]Lu-DOTA-r-a-Abz-analog (based on its in vitro characteristics) was attributed to a “sink effect” in mCXCR4-positive organs, especially the liver, due to the > 11-fold higher mCXCR4 affinity of [^177^Lu]Lu-DOTA-r-a-Abz-*iodo*CPCR4 compared to [^177^Lu]Lu-PentixaTher.

To confirm if this is also the case for [^99m^Tc]Tc-N_4_-**L6**-CPCR4, we additionally determined its mCXCR4 affinity in HEK cells stably transduced with mCXCR4, using [^125^I]CPCR4.3 as radioligand. Compared to previous experiments using mCXCR4 overexpressing Eµ-myc 1080 mouse lymphoma cells [24, 33], the absolute IC_50_ value for the reference ^nat^Lu-PentixaTher in the new experimental setup was fivefold higher (IC_50_ = 2568 ± 114 nM vs 567 ± 62 nM, respectively). With an IC_50_ of 66.4 ± 16.3 nM, N_4_-**L6**-CPCR4 has thus an almost 40-fold higher mCXCR4 affinity than ^nat^Lu-PentixaTher, which is even higher than that of the [^177^Lu]Lu-DOTA-r-a-Abz-ligand cited above [24]. This corroborates the assumption that a significant proportion of [^99m^Tc]Tc-N_4_-**L6**-CPCR4 in the lung, liver, and spleen is mCXCR4 specific, as has also been demonstrated the a blocking experiment (coinjection of 50 µg AMD3100) shown in Fig. 2. Thus, importantly, the observed high liver uptake of [^99m^Tc]Tc-N_4_-**L6**-CPCR4 in mice will most probably not be predictive for the human situation, because hCXCR4 is not expressed in the normal liver [44].

Despite this non-negligible on-target-off-site accumulation of [^99m^Tc]Tc-N_4_-**L6**-CPCR4, however, its tumor-to-background ratios for all non-target organs are comparable to those obtained for the more hydrophilic analog [^99m^Tc]Tc-HYNIC-**L6**-CPCR4 (Supplementary Fig. 1), and the observed minor differences are statistically not significant. Thus, since [^99m^Tc]Tc-N_4_-**L6**-CPCR4 showed the highest CXCR4 affinity, internalization, and tumor accumulation of the novel ^99m^Tc-labeled CPCR4-analogs in this study and was reliably obtained in high radiochemical yields using a kit-like procedure, [^99m^Tc]Tc-N_4_-**L6**-CPCR4 (termed [^99m^Tc]Tc-PentixaTec for the remainder of the manuscript) was selected as lead compound for clinical translation.

### In vitro and in vivo stability of [^99m^Tc]Tc-PentixaTec

In preparation for the early translation to the human setting, in vitro and in vivo stability of [^99m^Tc]Tc-PentixaTec in various media was assessed (see Supplementary Fig. 2). Only when diluted with saline and under challenging conditions (citrate and DTPA), progressive decomposition of the radiopharmaceutical was observed. In contrast, [^99m^Tc]Tc-PentixaTec was found to be fully stable in all physiological media (whole blood, serum, human serum albumin (2.5 and 5%)) over an extended period of time (13 h). In the original product formulation, the percentage of intact [^99m^Tc]Tc-PentixaTec slowly decreased from 94.5 to 90.6% within 2.5 h, suggesting that the tracer should ideally be administered within 2 h after the end of synthesis to ensure maximum radiochemical purity of the tracer.

### Proof-of-concept clinical [^99m^Tc]Tc-PentixaTec SPECT/CT imaging

In a next step, CXCR4-directed scintigraphy with [^99m^Tc]Tc-PentixaTec was performed in five patients with a history of hematologic disease. Out of those, four individuals underwent dedicated dosimetry. Imaging was well tolerated and no adverse events were recorded.

**Fig. 3:**
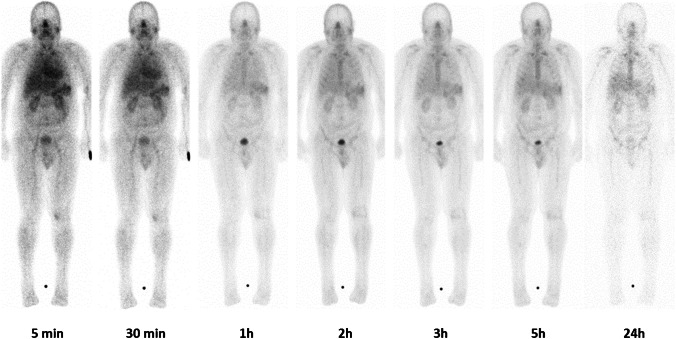
Biodistribution and clearance kinetics of [^99m^Tc]Tc-PentixaTec in a patient with a history of multiple myeloma and a suspicious finding in the right tibia in [^18^F]FDG PET/CT. CXCR4-directed whole-body scintigraphy is unremarkable, a subsequent biopsy of the lesion ruled out multiple myeloma

**Fig. 4:**
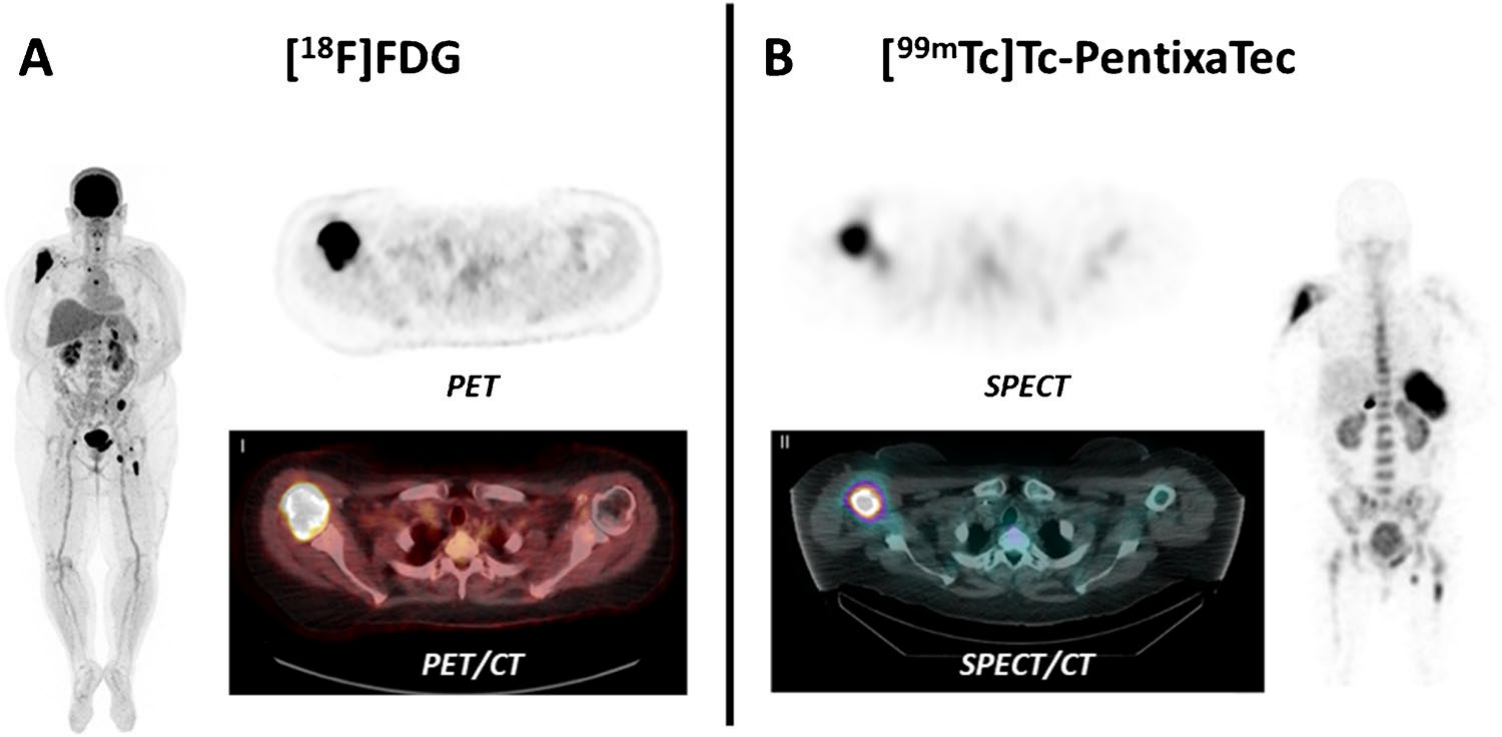
Comparison of [^18^F]FDG-PET/CT (**A**) and [^99m^Tc]Tc-PentixaTec (**B**) SPECT/CT 4 h p.i. in a patient with B-NHL. Whereas transaxial slices of the lymphoma manifestation in the right humerus depict intense CXCR4 expression, other lesions (left os ilium and right infraclavicular lymph nodes) are receptor-negative as an example of intra-individual tumor heterogeneity

In all patients, [^99m^Tc]Tc-PentixaTec was rapidly cleared from the blood pool with a median plasma half-life of 19 min (range 15–25 min, see Supplementary Fig. 3) and exhibited a very favorable in vivo distribution with no relevant background accumulation. Of note, as compared to [^99m^Tc]Tc-CPCR4-L, [^99m^Tc]Tc-PentixaTec demonstrated no significant hepatobiliary excretion. An example of clearance kinetics and normal biodistribution of [^99m^Tc]Tc-PentixaTec over 24 h is depicted in Fig. 3. Figure 4 provides an illustrative comparison between [^18^F]FDG-PET/CT and [^99m^Tc]Tc-PentixaTec scintigraphy/SPECT/CT in a patient with B cell non-Hodgkin’s lymphoma who underwent chemokine receptor imaging for assessment of potential CXCR4-directed radioligand therapy.

### [^99m^Tc]Tc-PentixaTec patient dosimetry

Table 4 summarizes the calculated absorbed organ doses for four patients (the remaining subject with B-NHL did not undergo dedicated dosimetry). The spleen as a pool for CXCR4 expressing blood cells received the highest absorbed dose with a median value of 1.95E − 02 mGy/MBq (range, 1.61E − 02–2.38E − 02), followed by the kidneys (median 1.23E − 02 mGy/MBq; range, 0.94 E − 02–1.37E − 02) and bone marrow (median 9.40E − 03 mGy/MBq; range, 6.2E − 03–13.50E − 03). The median effective dose per unit of administered activity was 5.3E − 03 mSv/MBq (range, 4.3E − 03–6.7E − 03) and thus almost equal to the numbers reported for [^99m^Tc]CXCR4-L (3.92E − 03 mSv/MBq [22]).

**Table 4:** Absorbed organ doses (mGy) per 1 MBq of [^99m^Tc]Tc-PentixaTec

Organ	Patient 1	Patient 2	Patient 3	Patient 4	Median	Range	
						*Min*	*Max*
Adrenals	0.0053	0.0078	0.0083	0.00524	0.0065	0.0052	0.0083
Brain	0.0023	0.0032	0.0039	0.00278	0.0030	0.0023	0.0039
Breast	0.0021	0.0028	0.0032	0.00227	0.0025	0.0021	0.0032
Gallbladder wall	0.0051	0.0068	0.0073	0.00502	0.0060	0.0050	0.0073
LLI wall	0.0037	0.0053	0.0062	0.00428	0.0048	0.0037	0.0062
Small intestine	0.0041	0.0053	0.0062	0.00455	0.0049	0.0041	0.0062
Stomach wall	0.0040	0.0057	0.0066	0.00441	0.0051	0.0040	0.0066
ULI wall	0.0040	0.0055	0.0063	0.00441	0.0049	0.0040	0.0063
Heart wall	0.0040	0.0049	0.0054	0.00317	0.0044	0.0032	0.0054
Kidneys	0.0137	0.0117	0.0128	0.00936	0.0123	0.0094	0.0137
Liver	0.0066	0.0087	0.0089	0.00636	0.0077	0.0064	0.0089
Lungs	0.0051	0.0079	0.0075	0.00337	0.0063	0.0034	0.0079
Muscle	0.0028	0.0038	0.0044	0.00318	0.0035	0.0028	0.0044
Ovaries	0.0040	0.0054	0.0064	0.00451	0.0050	0.0040	0.0064
Pancreas	0.0055	0.0083	0.0092	0.00571	0.0070	0.0055	0.0092
Red marrow	0.0065	0.0135	0.0123	0.00617	0.0094	0.0062	0.0135
Osteogenic cells	0.0099	0.0162	0.0184	0.011	0.0136	0.0099	0.0184
Skin	0.0018	0.0024	0.0027	0.00209	0.0023	0.0018	0.0027
Spleen	0.0161	0.0238	0.0202	0.0188	0.0195	0.0161	0.0238
Testes	0.0023			0.00282	0.0026	0.0023	0.0028
Thymus	0.0030	0.0040	0.0045	0.00324	0.0036	0.0030	0.0045
Thyroid	0.0028	0.0032	0.0039	0.00329	0.0033	0.0028	0.0039
Urinary bladder wall	0.0033	0.0043	0.0053	0.00385	0.0041	0.0033	0.0053
Uterus	0.0038	0.0049	0.0059	0.0044	0.0047	0.0038	0.0059
Total body	0.0028	0.0039	0.0052	0.00351	0.0037	0.0028	0.0052

Injection of a standard activity of 500 MBq [^99m^Tc]Tc-PentixaTec resulted in a median effective dose of 2.7 mSv (range, 2.1–3.4). Therefore, [^99m^Tc]Tc-PentixaTec compares favorably to established PET agents such as [^68^ Ga]Ga-PentixaFor or [^68^ Ga]Ga-NOTA-NFB for which imaging with a standard activity of 150 MBq has been reported to result in effective doses of 2.3 mSv [45] and 3.8 mSv [46], respectively.

## Conclusion

In this study, both the role of the structural composition of the Abz-a-r-(aa_3_) linker, connecting the CPCR4 peptide core and the radiolabel, and the influence of the radiolabeling strategy (mas_3_ vs HYNIC vs N_4_) on the overall in vitro and in vivo characteristics of a given CPCR4-linker-construct (**L6**-CPCR4) were investigated in detail. Our results demonstrate that positioning a small cationic amino acid at the far end of the linker (aa_3_ = dap, **L6**) greatly improves CXCR4 affinity and the flexibility towards N-terminal structural modifications (different chelators) in CPCR4-Abz-a-r-aa_3_-based peptides. Of all compounds investigated, [^99m^Tc]Tc-N_4_-**L6**-CPCR4 ([^99m^Tc]Tc-PentixaTec) showed the most promising overall in vitro and in vivo characteristics, combining a robust, clinically translatable radiolabeling chemistry, high in vitro stability, a suitable lipophilicity, excellent CXCR4 targeting and internalization, both in vitro and in vivo, and an appropriate pharmacokinetic profile, allowing high-contrast visualization of CXCR4 expressing tissues at early time points. In a first proof-of-concept human application in a group of five patients with hematological cancers, [^99m^Tc]Tc-PentixaTec was well tolerated, allowed high-contrast delineation of CXCR4 expressing tumors, and exhibited a favorable overall biodistribution and dosimetry profile. Further clinical research to consolidate the value of [^99m^Tc]Tc-PentixaTec SPECT/planar imaging as an alternative modality to CXCR4-targeted PET is therefore highly warranted.

### Supplementary Information

Below is the link to the electronic supplementary material.

Supplementary file1 (DOCX 406 kb)

## Data Availability

The datasets generated during and/or analyzed during the current study are available from the corresponding authors on reasonable request.

## References

[1] Wester HJ, Keller U, Schottelius M, Beer A, Philipp-Abbrederis K, Hoffmann F (2015). Disclosing the CXCR4 expression in lymphoproliferative diseases by targeted molecular imaging. Theranostics.

[2] Philipp-Abbrederis K, Herrmann K, Knop S, Schottelius M, Eiber M, Luckerath K (2015). In vivo molecular imaging of chemokine receptor CXCR4 expression in patients with advanced multiple myeloma. EMBO Mol Med.

[3] Lapa C, Schreder M, Schirbel A, Samnick S, Kortum KM, Herrmann K (2017). [(68)Ga]Pentixafor-PET/CT for imaging of chemokine receptor CXCR4 expression in multiple myeloma - comparison to [(18)F]FDG and laboratory values. Theranostics.

[4] Duell J, Krummenast F, Schirbel A, Klassen P, Samnick S, Rauert-Wunderlich H (2021). Improved primary staging of marginal-zone lymphoma by addition of CXCR4-directed PET/CT. J Nucl Med.

[5] Buck AK, Haug A, Dreher N, Lambertini A, Higuchi T, Lapa C (2022). Imaging of C-X-C motif chemokine receptor 4 expression in 690 patients with solid or hematologic neoplasms using (68)Ga-pentixafor PET. J Nucl Med.

[6] Herrmann K, Schottelius M, Lapa C, Osl T, Poschenrieder A, Haenscheid H, et al. First-in-man experience of CXCR4-directed endoradiotherapy with 177Lu-and 90Y-labelled pentixather in advanced stage multiple myeloma with extensive intra-and extramedullary disease. J Nucl Med. 2015. 10.2967/jnumed.115.167361. 10.2967/jnumed.115.167361 26564323

[7] Lapa C, Hanscheid H, Kircher M, Schirbel A, Wunderlich G, Werner RA (2019). Feasibility of CXCR4-directed radioligand therapy in advanced diffuse large B-cell lymphoma. J Nucl Med.

[8] Lapa C, Herrmann K, Schirbel A, Hanscheid H, Luckerath K, Schottelius M (2017). CXCR4-directed endoradiotherapy induces high response rates in extramedullary relapsed multiple myeloma. Theranostics.

[9] Borchert T, Beitar L, Langer LBN, Polyak A, Wester HJ, Ross TL (2019). Dissecting the target leucocyte populations of clinically relevant inflammation radiopharmaceuticals. J Nucl Cardiol.

[10] Bouter C, Meller B, Sahlmann CO, Staab W, Wester HJ, Kropf S (2018). (68)Ga-pentixafor PET/CT imaging of chemokine receptor CXCR4 in chronic infection of the bone: first insights. J Nucl Med.

[11] Derlin T, Gueler F, Brasen JH, Schmitz J, Hartung D, Herrmann TR (2017). Integrating MRI and chemokine receptor CXCR4-targeted PET for detection of leukocyte infiltration in complicated urinary tract infections after kidney transplantation. J Nucl Med.

[12] Lapa C, Reiter T, Werner RA, Ertl G, Wester HJ, Buck AK (2015). [(68)Ga]Pentixafor-PET/CT for imaging of chemokine receptor 4 expression after myocardial infarction. JACC Cardiovasc Imaging.

[13] Schmid JS, Schirbel A, Buck AK, Kropf S, Wester HJ, Lapa C (2016). [68Ga]Pentixafor-positron emission tomography/computed tomography detects chemokine receptor CXCR4 expression after ischemic stroke. Circ Cardiovasc Imaging.

[14] Thackeray JT, Derlin T, Haghikia A, Napp LC, Wang Y, Ross TL (2015). Molecular imaging of the chemokine receptor CXCR4 after acute myocardial infarction. JACC Cardiovasc Imaging.

[15] Li X, Yu W, Wollenweber T, Lu X, Wei Y, Beitzke D (2019). [(68)Ga]Pentixafor PET/MR imaging of chemokine receptor 4 expression in the human carotid artery. Eur J Nucl Med Mol Imaging.

[16] Buck AK, Serfling SE, Lindner T, Hänscheid H, Schirbel A, Hahner S (2022). CXCR4-targeted theranostics in oncology. Eur J Nucl Med Mol Imaging.

[17] Kircher M, Herhaus P, Schottelius M, Buck AK, Werner RA, Wester HJ (2018). CXCR4-directed theranostics in oncology and inflammation. Ann Nucl Med.

[18] Schottelius M, Herrmann K, Lapa C (2021). In vivo targeting of CXCR4-new horizons. Cancers (Basel).

[19] Zhang X, You L, Chen S, Gao M, Guo Z, Du J (2018). Development of a novel (99m) Tc-labeled small molecular antagonist for CXCR4 positive tumor imaging. J Labelled Comp Radiopharm.

[20] Mikaeili A, Erfani M, Shafiei M, Kobarfard F, Abdi K, Sabzevari O (2018). Development of a (99m)Tc-labeled CXCR4 antagonist derivative as a new tumor radiotracer. Cancer Biother Radiopharm.

[21] Avila-Sanchez M, Ferro-Flores G, Jimenez-Mancilla N, Ocampo-Garcia B, Bravo-Villegas G, Luna-Gutierrez M (2020). Synthesis and preclinical evaluation of the Tc-99m-/Lu-177-CXCR4-L theranostic pair for in vivo chemokine-4 receptor-specific targeting. J Radioanal Nucl Chem.

[22] Vallejo-Armenta P, Santos-Cuevas C, Soto-Andonaegui J, Villanueva-Perez RM, Gonzalez-Diaz JI, Garcia-Perez FO (2020). (99m)Tc-CXCR4-L for imaging of the chemokine-4 receptor associated with brain tumor invasiveness: biokinetics, radiation dosimetry, and proof of concept in humans. Contrast Media Mol Imaging.

[23] Demmer O, Gourni E, Schumacher U, Kessler H, Wester HJ (2011). PET imaging of CXCR4 receptors in cancer by a new optimized ligand. ChemMedChem.

[24] Osl T, Schmidt A, Schwaiger M, Schottelius M, Wester HJ (2020). A new class of PentixaFor- and PentixaTher-based theranostic agents with enhanced CXCR4-targeting efficiency. Theranostics.

[25] Poschenrieder A, Schottelius M, Schwaiger M, Kessler H, Wester HJ (2016). The influence of different metal-chelate conjugates of pentixafor on the CXCR4 affinity. EJNMMI Res.

[26] Banerjee SR, Maresca KP, Francesconi L, Valliant J, Babich JW, Zubieta J (2005). New directions in the coordination chemistry of 99mTc: a reflection on technetium core structures and a strategy for new chelate design. Nucl Med Biol.

[27] Demmer O, Frank AO, Hagn F, Schottelius M, Marinelli L, Cosconati S (2012). A conformationally frozen peptoid boosts CXCR4 affinity and anti-HIV activity. Angew Chem Int Ed Engl.

[28] Robu S, Schottelius M, Eiber M, Maurer T, Gschwend J, Schwaiger M (2017). Preclinical evaluation and first patient application of 99mTc-PSMA-I&S for SPECT imaging and radioguided surgery in prostate cancer. J Nucl Med.

[29] Kuzmanovska S, Vaskova O, Zdraveska KM (2011). “In-house” preparation of 99mTc-EDDA/HYNIC-TOC, a specific targeting agent for somatostatin receptor scintigraphy. Maced Pharm Bull.

[30] Nock B, Nikolopoulou A, Chiotellis E, Loudos G, Maintas D, Reubi JC (2003). [99mTc]Demobesin 1, a novel potent bombesin analogue for GRP receptor-targeted tumour imaging. Eur J Nucl Med Mol Imaging.

[31] Weineisen M, Simecek J, Schottelius M, Schwaiger M, Wester HJ (2014). Synthesis and preclinical evaluation of DOTAGA-conjugated PSMA ligands for functional imaging and endoradiotherapy of prostate cancer. EJNMMI Res.

[32] Schottelius M, Osl T, Poschenrieder A, Herrmann K, Lapa C, Hoffmann F (2015). [177]Lu-pentixather: preclinical and first patient results with a highly promising CXCR4-directed endoradiotherapeutic agent. J Nucl Med.

[33] Schottelius M, Ludescher M, Richter F, Kapp TG, Kessler H, Wester HJ (2019). Validation of [(125)I]CPCR4.3 as an investigative tool for the sensitive and specific detection of hCXCR4 and mCXCR4 expression in vitro and in vivo. EJNMMI Res..

[34] Schottelius M, Simecek J, Hoffmann F, Willibald M, Schwaiger M, Wester HJ (2015). Twins in spirit - episode I: comparative preclinical evaluation of [(68)Ga]DOTATATE and [(68)Ga]HA-DOTATATE. EJNMMI Res.

[35] Kletting P, Schimmel S, Kestler HA, Hanscheid H, Luster M, Fernandez M (2013). Molecular radiotherapy: the NUKFIT software for calculating the time-integrated activity coefficient. Med Phys.

[36] Stabin MG, Sharkey RM, Siegel JA (2011). RADAR commentary: evolution and current status of dosimetry in nuclear medicine. J Nucl Med.

[37] Siegel JA, Thomas SR, Stubbs JB, Stabin MG, Hays MT, Koral KF (1999). MIRD pamphlet no. 16: techniques for quantitative radiopharmaceutical biodistribution data acquisition and analysis for use in human radiation dose estimates. J Nucl Med..

[38] The 2007 recommendations of the International Commission on Radiological Protection. ICRP publication 103. Ann ICRP. 2007;37*:*1–332. 10.1016/j.icrp.2007.10.003 18082557

[39] Mecozzi S, West AP, Dougherty DA (1996). Cation-pi interactions in aromatics of biological and medicinal interest: electrostatic potential surfaces as a useful qualitative guide. Proc Natl Acad Sci USA.

[40] Abiraj K, Mansi R, Tamma ML, Forrer F, Cescato R, Reubi JC (2010). Tetraamine-derived bifunctional chelators for technetium-99m labelling: synthesis, bioconjugation and evaluation as targeted SPECT imaging probes for GRP-receptor-positive tumours. Chemistry.

[41] Rao TN, Adhikesavalu D, Camerman A, Fritzberg AR (1991). Synthesis and characterization of monooxorhenium(V) complexes of mercaptoacetylglycylglycylglycine - crystal-structure of tetrabutylammonium oxo(mercaptoacetylglycylglycylglycine)rhenate(V). Inorg Chim Acta.

[42] Papadopoulos MS, Pirmettis IC, Pelecanou M, Raptopoulou CP, Terzis A, Stassinopoulou CI (1996). Syn-anti isomerism in a mixed-ligand oxorhenium complex, ReO[SN(R)S][S]. Inorg Chem.

[43] Frodl R, Gierschik P, Moepps B (1998). Genomic organization and expression of the CXCR4 gene in mouse and man: absence of a splice variant corresponding to mouse CXCR4-B in human tissues. J Recept Signal Transduct Res.

[44] Hu F, Miao L, Zhao Y, Xiao YY, Xu Q (2015). A meta-analysis for C-X-C chemokine receptor type 4 as a prognostic marker and potential drug target in hepatocellular carcinoma. Drug Des Devel Ther.

[45] Herrmann K, Lapa C, Wester HJ, Schottelius M, Schiepers C, Eberlein U (2015). Biodistribution and radiation dosimetry for the chemokine receptor CXCR4-targeting probe 68Ga-pentixafor. J Nucl Med.

[46] Wang Z, Zhang M, Wang L, Wang S, Kang F, Li G (2015). Prospective study of (68)Ga-NOTA-NFB: radiation dosimetry in healthy volunteers and first application in glioma patients. Theranostics.

