# Supplementary material for: [99mTc]Tc-PentixaTec: development, extensive pre-clinical evaluation, and first human experience
Source: Eur J Nucl Med Mol Imaging. 2023 Aug 19;50(13):3937–48. doi: 10.1007/s00259-023-06395-x (PMC10611619; doi:10.1007/s00259-023-06395-x)
Supplement: Supplementary file 1 — Supplementary file1 (DOCX 406 kb) [file 259_2023_6395_MOESM1_ESM.docx]

**Supplemental information to**

**[^99m^Tc]Tc-PentixaTec: Development, extensive pre-clinical evaluation and first human experience**

Matthias Konrad^1^, Andreas Rinscheid^2^, Georgine Wienand^3^, Bernd Nittbaur^3^, Hans-Jürgen Wester^1^, Tilman Janzen^2^, Constantin Lapa^3^, Christian Helmut Pfob^3^, Margret Schottelius^4,5,6^

^1^ Chair for Pharmaceutical Radiochemistry, Faculties of Chemistry and Medicine, Technische Universität München, 85748 Garching, Germany

^2^ Medical Physics and Radiation Protection, University Hospital Augsburg, Stenglinstrasse 2, 86156 Augsburg, Germany

^3^ Nuclear Medicine, Faculty of Medicine, University of Augsburg, Stenglinstrasse 2, 86156 Augsburg, Germany

^4^ Translational Radiopharmaceutical Sciences, Department of Nuclear Medicine and Department of Oncology, Centre Hospitalier Universitaire Vaudois (CHUV) and University of Lausanne (UNIL), 1011 Lausanne, Switzerland

^5^ AGORA, Pôle de recherche sur le cancer, 1011 Lausanne, Switzerland

^6^ SCCL Swiss Cancer Center Leman, 1011 Lausanne, Switzerland

**General**

**Reagents and solvents**

Purchased reagents were used without further purification. The Fmoc-(9-fluorenylmethoxycarbonyl-) and all other protected amino acid analogs were purchased from Bachem (Bubendorf, Switzerland), Iris Biotech GmbH (Marktredwitz, Germany), Carbolution Chemicals GmbH (St. Ingbert, Germany) and Merck Millipore (Darmstadt, Germany). The 2-Chlorotrityl chloride (2-CTC) resin was obtained from Iris Biotech GmbH (Marktredwitz, Germany) or CEM (Matthews, USA). Reagents for peptide synthesis were purchased from Iris Biotech GmbH (Marktredwitz, Germany), Sigma-Aldrich (Munich, Germany) and Molekula GmBH (Garching, Germany). Solvents and reagents for organic synthesis were purchased from either Alfa Aesar (Karlsruhe, Germany), Sigma-Aldrich (Munich, Germany) or VWR (Darmstadt, Germany).

Water for RP-HPLC solvents was obtained from the in-house Millipore system from Thermo Fischer Scientific Inc. (Waltham MA, USA). Tracepure water for labeling experiments was purchased from Merck Millipore (Darmstadt, Germany).

**Instrumentation**

Solid-phase peptide synthesis (SPPS) was carried out by manual operation using an Intelli-Mixer syringe shaker from Neolab (Heidelberg, Germany).

Analytical and semi-preparative reversed-phase high performance liquid chromatography (RP-HPLC) was performed using a Shimadzu HPLC system (Shimadzu Deutschland GmbH, Neufahrn, Germany) equipped with a SPD-20A UV/Vis detector (λ = 220 nm, 254 nm). For analytical RP-HPLC, a Multokrom 100 C18 (125 × 4.6 mm, 5 μm particle size) column (CS Chromatographie Service GmbH, Langerwehe, Germany) was used at a flow rate of 1 mL/min. Semi-preparative HPLC purification was performed using a Multokrom 100 RP 18 (250 × 10 mm, 5 μm particle size) column (CS GmbH, Langerwehe, Germany) at a constant flow rate of 5 mL/min.

As mobile phase, various gradients of acetonitrile (0.1% TFA, solvent B) in water (0.1% TFA, solvent A) were used. Specific gradients are mentioned in the text.

Purification via flash-chromatography was carried out on a Isolera^TM^ Prime System from Biotage (Uppsala, Sweden), running a Biotage 09474 Rev. E Bio pump. A Biotage^TM^ SNAP KP-C_18_ cartridge (12 g, 93 Å pore diameter, 382 m^2^/g surface) was used applying a linear gradient of solvent B (ACN, 0.1 vol% TFA, 2 vol% H_2_O) in solvent A (H_2_O, 0.1 vol% TFA).

Radio-RP-HPLC was also performed using a Multokrom 100 C18 (125 × 4.6 mm, 5 μm particle size) column (CS Chromatographie Service GmbH, Langerwehe, Germany). For radioactivity detection, the outlet of the UV-photometer was connected to a NaI(Tl) well-type scintillation counter from EG&G Ortec (Munich, Germany). Radio-TLC measurements were conducted on a Scan-RAM^TM^ from LabLogic Sstems Ltd. (Broomhill, UK). TLC spectra were analyzed using the Laura^TM^ software from LabLogic Sstems Ltd. (Broomhill, UK).

Final products were lyophilized using an Alpha 1-2 LDplus lyophilization instrument from Christ (Osterode am Harz, Germany), connected to a RZ-2 vacuum pump from Vacubrand GmbH (Wertheim, Germany). Mass spectrometry analysis was carried out using an expression^L^ CMS quadrupole mass spectrometer from Advion Ltd. (Harlow, UK).

**Synthesis**

**CPCR4 cyclic pentapeptide backbone**

The synthesis of the CXCR4 binding motif CPCR4 was performed in analogy to a previously described procedure [1]. In short, Fmoc-Gly-OH was immobilized on 2-CTC resin, and Fmoc-2-Nal-OH, Fmoc-Arg(Pbf)-OH and Fmoc-D-Orn(Boc)-OH were coupled successively using a standard SPPS (solid phase peptide synthesis) Fmoc protocol with HOBt/TBTU as coupling reagents and DIPEA (*N,N*-diisopropylethylamine) as base. The *N*-terminus was then Fmoc deprotected and newly protected by reaction with NBS-Cl (2-Nitrobenzene sulfonylchloride, 4.00 eq.) and 2,4,6-Collidine (10.0 eq.) as base in NMP (N-Methyl-Pyrrolidon) for 15 min. N-Methylation of the NBS-protected amino terminus was then performed using Dimethylsulfate (10.0 eq.), DBU (1,8-Diazabicyclo[5.4.0]undec-7-en, 3.0 eq.) in NMP (2x2 min) [2]. Deprotection of the methylated terminus was achieved by incubation of the peptide resin with DBU (5.00 eq.) for 5 min, followed by the addition of 2-mercaptoethanol (10.00 eq.). After 30 min, the resin was washed thoroughly, and the subsequent coupling of Fmoc-D-Tyr(*t*Bu)-OH was carried out using HOAt and HATU as coupling reagents. After final Fmoc deprotection, the peptide was cleaved from the resin using a mixture of TFA/TIPS/H_2_O (95/2.5/2.5 (v/v/v)), leading to concomitant removal of all acid labile protecting groups. Upon concentration of the cleavage mixture, precipitation of the crude peptide using diethyl ether and drying, the peptide was redissolved in DMF to yield a 1 mM solution, and cyclization was carried out using DPPA (Diphenylphosphorylazide, 3.0 eq) and NaHCO_3_ (5.0 eq.) Purification of the peptide by flash-chromatography yielded an off-white solid.

CPCR4: RP-HPLC (10 – 95% B in 15 min): *t_R_* = 6.49 min. Calculated monoisotopic mass (C_36_H_47_N_9_O_6_): 701.36, found: 701.8 [M+H$]^{+}$, 351.4 [M+2H$]^{2+}$.

**Linker moieties**

All Abz (p-aminobenzoic acid )-based linkers or linker fragments used in this study were synthesized following the same general protocol, i.e. loading of 2-CTC-resin with Fmoc-Abz-OH, subsequent assembly of the respective peptide sequences on the solid support, and cleavage of the fully protected (including N-terminal Fmoc) products using a mixture of DCM/HFIP (4/1 (v/v), 8 mL/g resin).

Of note, for the attachment of the first amino acid to the relatively unreactive amino group of Abz, HOAt (1-Hydroxy-7-azabenzotriazol) and HATU (O-(7-Azabenzotriazol-1-yl)-N,N,N′,N′-tetramethyluronium-hexafluorphosphate) were used as coupling reagents, and reaction times were extended to 4h to achieve quantitative coupling. To avoid racemization of Fmoc-dap(Boc)-OH, DIPEA was replaced by 2,4,6-Collidine during coupling of Fmoc-dap(Boc)-OH.

Fmoc-r(Pbf)-a-Abz-OH (**L1**)


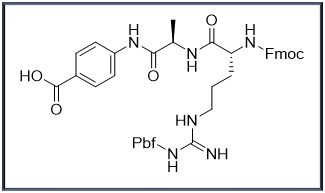
RP-HPLC (10-90% B in 15 min): *t_R_* = 15.33 min. Calculated monoisotopic mass (C_44_H_50_N_6_O_9_S): 838.34, found: 839.1 [M+H$]^{+}$.

Fmoc-dap(Boc)-r(Pbf)-a-Abz-OH (**L6**)


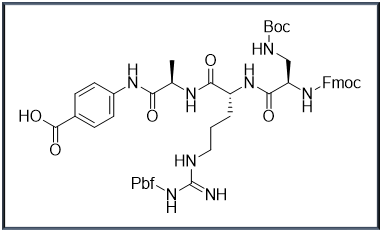
RP-HPLC (50-95% B in 15 min): *t_R_* = 13.52 min. Calculated monoisotopic mass (C_52_H_64_N_8_O_12_S): 1024.44, found: 1025.2 [M+H$]^{+}$.

**Tc-chelators**

(Trt)ma-(s(tBu))_3_-OH

The protected mas_3_-precursor was synthesized via SPPS as described for the linker moieties.

**
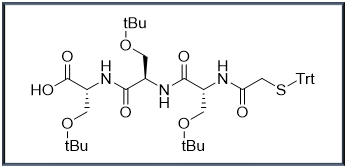
**RP-HPLC (10-90% B in 15 min): *t_R_* = 14.85 min. Calculated monoisotopic mass (C_42_H_57_N_3_O_8_S): 763.39, found: 764.7[M+H$]^{+}$.

Boc-HYNIC

Boc-protection of HYNIC (hydrazino-nicotinic acid) was performed in analogy to a published protocol [3, 4]. In short, HYNIC was reacted with Boc_2_O (1.0 eq.) and Triethylamine (1.3 eq.) in DMF overnight. The solvent was evaporated under reduced pressure and the crude product was isolated via silica flash-chromatography using EtOAc, followed by EtOAc + 1vol% AcOH, as eluents. The desired product was obtained as a white powder.

Calculated monoisotopic mass (C_11_H_15_N_3_O_4_): 253.11, found: 254.4 [M+H$]^{+}$.

(Boc)_4_N_4_-chelator


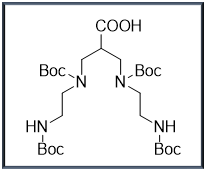
The fully Boc-protected N4-bifunctional chelator was synthesized according to a literature protocol [5]. Briefly, *tert*-butyl‑(2-aminoethyl)‑carbamate (4.0 eq.) was slowly added to 3‑bromo‑2‑(bromomethyl)-propanoic acid (1.0 eq.) in THF (25 mL/mmol) under vigorous stirring. The mixture was stirred for 4 h at ambient temperature before removing the solvent under reduced pressure at room temperature. The crude product was dissolved in acetone:H_2_O (1:1, 25 mL/mmol), cooled to 0 °C and NEt_3_ (3.0 eq.) was added. After 5 min preactivation, Boc_2_O (4.0 eq.) was added. The mixture was stirred for 15 h (0°C to r.t.), the solvent removed under reduced pressure and the raw product purified via flash chromatography (35-95 % MeCN in H_2_O, 15 min).

R_f_ (EtOAc+0.5%AcOH) = 0.65; calculated monoisotopic mass for C_28_H_52_N_4_O_10_: 604.37; found by ESI‑MS: m/z = 605.0 [M+H]^+^.

**Protected mas_3_-conjugates**

All protected mas_3_-conjugated synthons were synthesized via SPPS as described for the linker moieties.

(Trt)ma-(s(tBu))_3_-a-OH


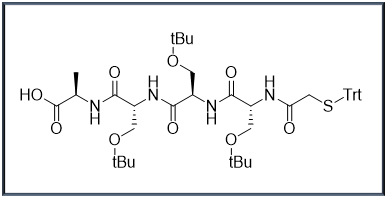
RP-HPLC (40-100% B in 15 min): *t_R_* = 16.54 min. Calculated monoisotopic mass (C_45_H_62_N_4_O_9_S): 834.42, found: 835.2[M+H$]^{+}$.

(Trt)ma-(s(tBu))_3_-dap(Boc)-OH


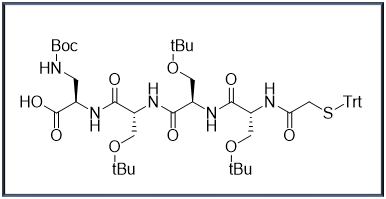
RP-HPLC (40-100% B in 15 min): *t_R_* = 17.60 min. Calculated monoisotopic mass (C_50_H_71_N_5_O_11_S): 949.49, found: 950.6[M+H$]^{+}$.

(Trt)ma-(s(tBu))_3_-r(Pbf)-r(Pbf)-a-Abz-OH


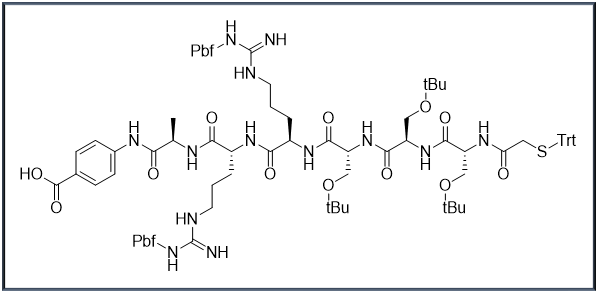
RP-HPLC (10-90% B in 15 min): *t_R_* = 18.41 min. Calculated monoisotopic mass (C_90_H_123_N_13_O_18_S_3_): 1769.83, found: 1771.2[M+H$]^{+}$.

(Trt)ma-(s(tBu))_3_-f-r(Pbf)-a-Abz-OH


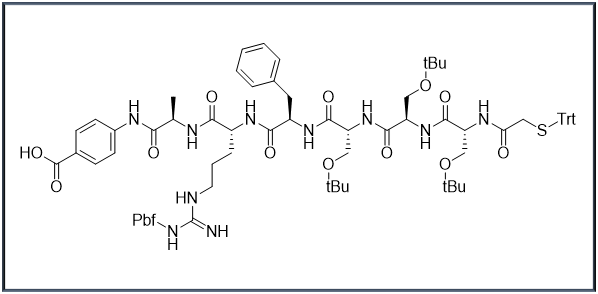
RP-HPLC (10-90% B in 15 min): *t_R_* = 17.44 min. Calculated monoisotopic mass (C_80_H_104_N_10_O_15_S_2_): 1508.71, found: 1509.8[M+H$]^{+}$.

(Trt)ma-(s(tBu))_3_-h(Trt)-r(Pbf)-a-Abz-OH


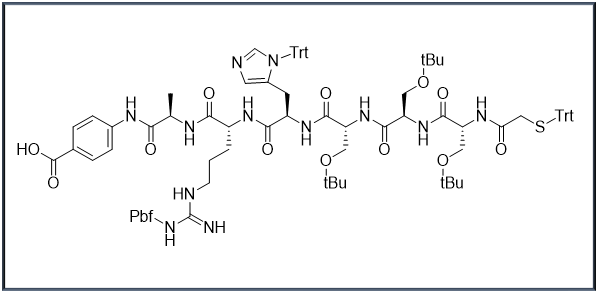
RP-HPLC (10-90% B in 15 min): *t_R_* = 17.80 min. Calculated monoisotopic mass (C_96_H_116_N_12_O_15_S_2_): 1740.81, found: 1742.3[M+H$]^{+}$.

**CPCR4-linker-chelator-conjugates (one-step fragment condensation)**

All CPCR4-linker conjugates were synthesized by fragment condensation between CPCR4 (1.0 eq.) and the respective Fmoc-protected linker (1.1 – 1.3 eq.), using HOAt/HATU as coupling reagents. If the activated amino acid was dap, 2,4,6-Collidine was used as base, in every other case DIPEA. Reaction mixtures were concentrated *in vacuo* before final cleavage of acid-labile protecting groups using TFA/TIPS/H_2_O (95/2.5/2.5 (v/v/v)). The crude products were then purified via semi-preparative HPLC.

mas_3_-**L3**-CPCR4

The product was obtained by fragment condensation of (Trt)ma-(s(tBu))_3_-r(Pbf)-r(Pbf)-a-Abz-OH with CPCR4 and subsequent deprotection.


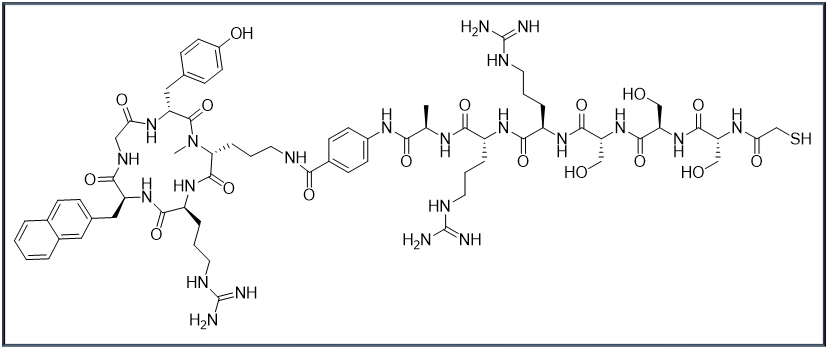
RP-HPLC (10-90% B in 15 min): *t_R_* = 9.00 min.

Calculated monoisotopic mass (C_69_H_98_N_22_O_17_S): 1538.72, found: 770.4[M+2H$]^{2+}$.

mas_3_-**L4**-CPCR4

The product was obtained by fragment condensation of (Trt)ma-(s(tBu))_3_-f-r(Pbf)-a-Abz-OH with CPCR4 and subsequent deprotection.


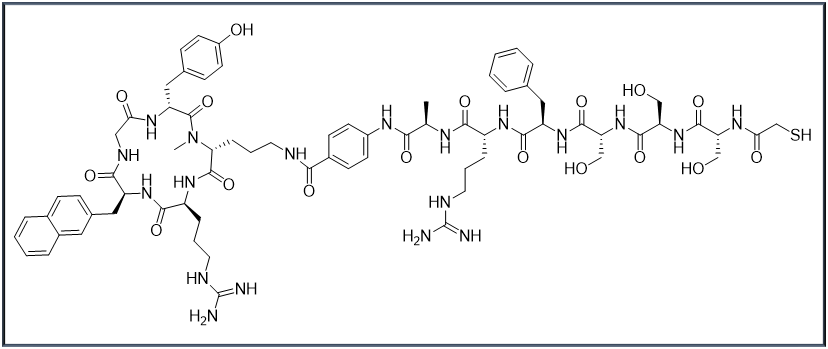
RP-HPLC (10-90% B in 15 min): *t_R_* = 10.20 min.

Calculated monoisotopic mass (C_72_H_95_N_19_O_17_S): 1529.69, found: 766.1[M+2H$]^{2+}$.

mas_3_-**L5**-CPCR4

The product was obtained by fragment condensation of (Trt)ma-(s(tBu))_3_-h(Trt)-r(Pbf)-a-Abz-OH with CPCR4 and subsequent deprotection.


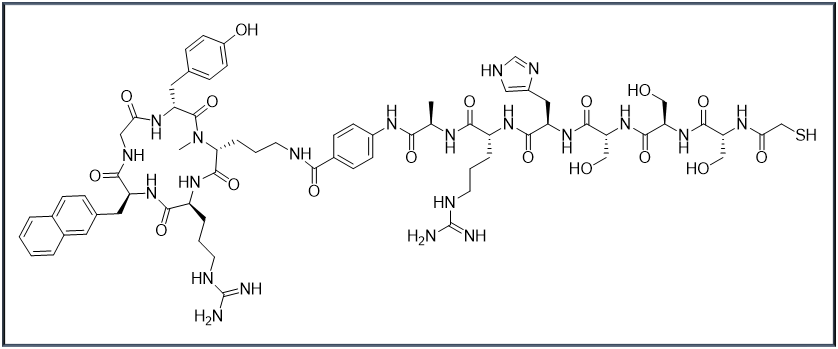
RP-HPLC (10-90% B in 15 min): *t_R_* = 9.53 min.

Calculated monoisotopic mass (C_69_H_93_N_21_O_17_S): 1519.68, found: 761.2[M+2H$]^{2+}$.

**CPCR4-linker-chelator-conjugates (two-step fragment condensation)**

All compounds in this section were obtained by successive conjugation

a) of CPCR4 with Fmoc-r(Pbf)-a-Abz-OH, followed by Fmoc-deprotection using 20% piperidine in DMF (v/v), and

b) of the resulting CXCR4-Abz-a-r(Pbf)-NH_2_ with either a Tc-chelator or one of the amino-acid-chelator conjugates.

Upon final cleavage of acid-labile protecting groups using TFA/TIPS/H_2_O (95/2.5/2.5 (v/v/v)), the crude products were purified via semi-preparative HPLC.

mas_3_-**L1**-CPCR4

The product was obtained by fragment condensation of (Trt)ma-(s(tBu))_3_-OH with CPCR4-Abz-a-r(Pbf)-NH_2_ and subsequent deprotection.


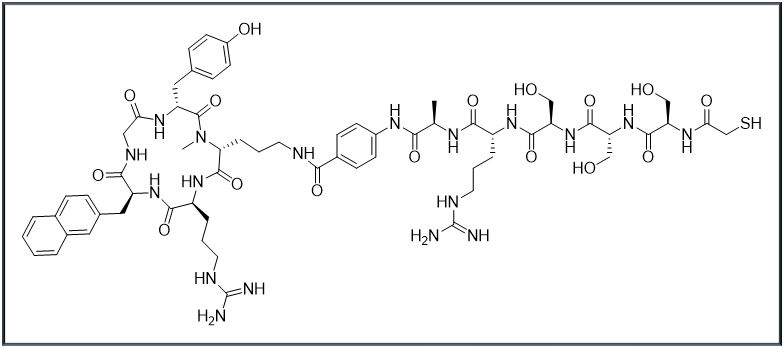
RP-HPLC (10-90% B in 15 min): *t_R_* = 9.82 min.

Calculated monoisotopic mass (C_63_H_86_N_18_O_16_S): 1382.62, found: 692.2[M+2H$]^{2+}$.

mas_3_-**L2**-CPCR4

The product was obtained by fragment condensation of (Trt)ma-(s(tBu))_3_-a-OH with CPCR4-Abz-a-r(Pbf)-NH_2_ and subsequent deprotection.


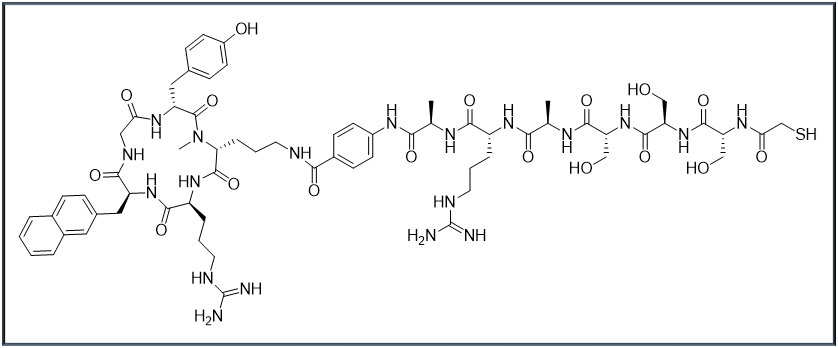
RP-HPLC (15-45% B in 15 min): *t_R_* = 9.10 min.

Calculated monoisotopic mass (C_66_H_91_N_19_O_17_S): 1453.66, found: 728.6[M+2H$]^{2+}$.

mas_3_-**L6**-CPCR4

The product was obtained by fragment condensation of (Trt)ma-(s(tBu))_3_-dap(Boc)-OH with CPCR4-Abz-a-r(Pbf)-NH_2_ and subsequent deprotection.


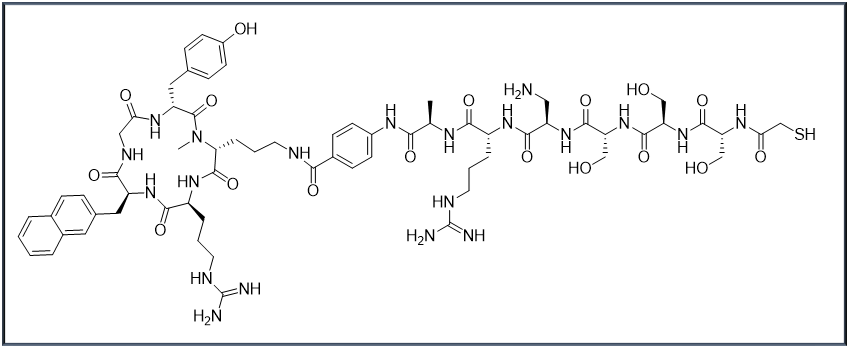
RP-HPLC (35-65% B in 15 min): *t_R_* = 8.85 min.

Calculated monoisotopic mass (C_66_H_92_N_20_O_17_S): 1468.67, found: 735.1[M+2H$]^{2+}$.

CPCR4-Abz-a-r(Pbf)-dap(Boc)-NH_2_ (**L6**-CPCR4)

The product was obtained by fragment condensation of Fmoc-dap(Boc)-r(Pbf)-a-Abz-OH (**L6**) with CPCR4 and subsequent deprotection.


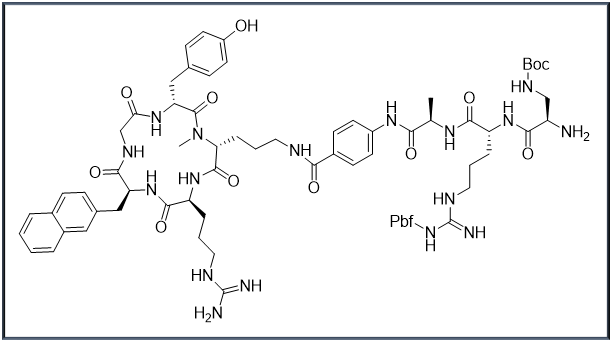
RP-HPLC (10-95% B in 15 min): *t_R_* = 8.83 min. Calculated monoisotopic mass (C_73_H_99_N_17_O_15_S): 1485.72, found: 744.6[M+2H$]^{2+}$.

HYNIC-**L6**-CPCR4

The product was obtained by fragment condensation of **L6**-CPCR4 with Boc-HYNIC and subsequent deprotection.


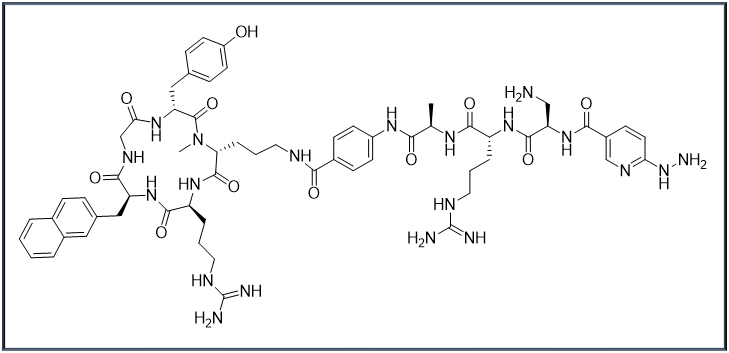
RP-HPLC (5-55% B in 15 min): *t_R_* = 7.88 min.

Calculated monoisotopic mass (C_61_H_80_N_20_O_11_): 1268.63, found: 635.6[M+2H$]^{2+}$.

N_4_-**L6**-CPCR4 (PentixaTec)

The product was obtained by fragment condensation of **L6**-CPCR4 with the (Boc)_4_N_4_-chelator and subsequent deprotection.


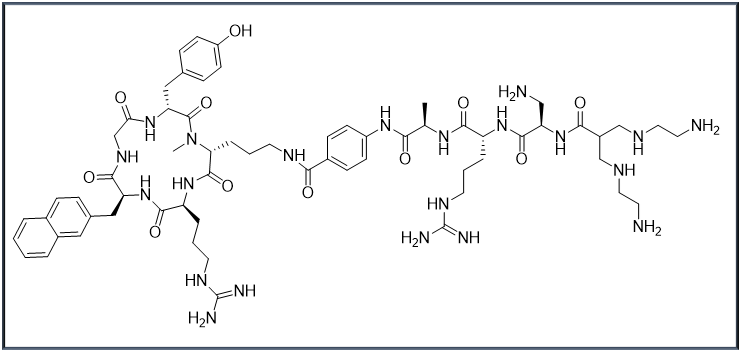
RP-HPLC (10–60% B in 15 min): *t_R_* = 8.03 min.

Calculated monoisotopic mass (C_63_H_93_N_21_O_11_): 1319.74, found: 660.7[M+2H$]^{2+}$, 441.0[M+3H$]^{3+}$.

**Radiolabeling**

**Radioiodination of FC-131 and CPCR4.3**

As previously reported [6], approximately 50-150 µg of unlabeled peptide recursor were dissolved in 20 µL DMSO, and added to 280 µL TRIS buffer (25 mM TRIS-HCl, 0.40 mM NaCl, pH = 7.5) in a Iodogen^®^-coated reaction tube. After addition of 5 µL [^125^I]NaI (15 – 20 MBq; Hartmann Analytics, Brauschweig, Germany), the mixture was incubated for 15 min at r.t.. The solution was then removed from the insoluble oxidant, and the radioiodinated product was isolated via RP-HPLC purification.

[^125^I]FC-131: RP-HPLC (20-55% B in 15 min): *t_R_* = 9.35 min.

[^125^I]CPCR4.3: RP-HPLC (25-40% B in 15 min): *t_R_* = 10.32 min

**^99m^Tc-labeling**

[^99m^Tc]-Pertechnetate was obtained by elution of a Drytech^TM^ Technetium Generator from GE Healthcare (Munich, Germany) with physiological NaCl solution (0.9%).

^99m^Tc-labeling of mas_3_-**L1-6**-CPCR4

For ^99m^Tc-labeling of all mas_3_-conjugated peptides, freeze-dried labeling kits were prepared in analogy to a previously employed protocol [7], containing 5 nmol peptide precursor, 666 μg sodium phosphate dibasic dihydrate, 29 μg disodium phosphate monobasic monohydrate, 2 mg Disodium tartrate dihydrate, 6 μg ascorbic acid and 8 μg Tin(II)chloride.

For ^99m^Tc-labeling, freshly eluted ^99m^Tc-Pertechnetate (0.10 – 5.00 mL, 50.0 – 850 MBq) was added, and the mixture was heated for 20 min at 90°C. Quality control of the reaction mixture was performed by radio-TLC using Silica-coated 60 RP-18 F_254_ strips (Merck Millipore, Darmstadt, Germany).

*Mobile phase NH_4_OAc/DMF (1/1, v/v):* R_f_ (^99m^TcO_4_^−^) = 1, R_f_ (^99m^Tc-colloid) = 0, R_f_ (^99m^Tc-tartrate) = 0.5-0.8, R_f_ (^99m^Tc-peptide) = 0.8 -1.

*Mobile phase 2-Butanone:* R_f_ (^99m^TcO_4_^−^) = 1, R_f_ (^99m^Tc-colloid) = 0, R_f_ (^99m^Tc-tartrate) = 0, R_f_ (^99m^Tc-peptide) = 0.

*Mobile phase NaCl (25 vol% in H_2_O)*: R_f_ (^99m^TcO_4_^−^) = 0, R_f_ (^99m^Tc-colloid) = 1, R_f_ (^99m^Tc-tartrate) = 0.5-0.8, R_f_ (^99m^Tc-peptide) = 0.

^99m^Tc-labeling of HYNIC-**L6**-CPCR4

Prior to labeling, freeze-dried kits were prepared according to a literature protocol [8] from the following solutions: 5 nmol peptide precursor in 5 μL DMSO/water, 50 μg EDDA (Ethylene diamine diacetic acid) in 50 μL 0.1 N NaOH, 2 mg disodium tartrate dihydrate in 50 μL NaH_2_PO_4_ buffer (40.0 g/L), and 8 μg Tin(II)chloride in aqueous sodium ascorbate (3.00 g/L in 0.01 M HCl).

For ^99m^Tc-labeling, freshly eluted ^99m^Tc-Pertechnetate (0.10 – 5.00 mL, 50.0 – 850 MBq) was added, and the mixture was heated for 20 min at 90°C. Quality control of the reaction mixture was performed by radio-TLC using Silica-coated 60 RP-18 F_254_ strips (Merck Millipore, Darmstadt, Germany).

Since radiochemical yields of [^99m^Tc]Tc-HYNIC-**L6**-CPCR4 were limited to max. 50%, the tracer was purified using SPE. Upon cooling, the reaction mixture was diluted with deionized water to a total volume of 10 mL and passed through a SepPak C18 plus (Waters, Eschborn, Germany) cartridge (preconditioned with 5 mL ethanol and 5 mL deionized water). The cartridge was then washed with 6 mL of deionized water, and dried with air. For elution, 1 mL of ethanol (0.5% AcOH) was used, and fractions of 3-4 drops of eluate were collected in Eppendorf vials. The fractions containing the highest amount of activity were combined, evaporated to dryness at 80°C under a nitrogen stream and reconstituted to the required activity concentration for the respective experiment using PBS.

^99m^Tc-labeling of N_4_-**L6**-CPCR4 (PentixaTec)

Prior to labeling, freeze-dried kits were prepared. Their composition was based on a previously published protocol [5] with slight adaptations. For ^99m^Tc-labeling, freshly eluted ^99m^Tc-Pertechnetate (0.10 – 5.00 mL, 50.0 – 850 MBq) was added, and the mixture was heated for 10 min at 95°C. Quality control of the reaction mixture was performed by radio-TLC using Silica-coated 60 RP-18 F_254_ strips (Merck Millipore, Darmstadt, Germany).

For patient application, the reaction mixture was diluted with saline (0.9% NaCl) to a final volume of 10 ml, pulled up into a syringe and passed through a Cathivex®-GV vented sterile filter unit (Merck Millipore, Darmstadt, Germany) into a sterilized glass vial before use.

**In vitro experiments**

**Cell culture**

CXCR4-positive Jurkat T lymphocyte cells were grown in RPMI 1640 GlutaMAX medium supplemented with 10 vol% FCS. Chem-1 cells stably transduced with hCXCR4 (ChemiScreen™ CXCR4 Receptor Stable Cell Line, eurofins DiscoveRx, Fremont, USA) were cultured in DMEM-F12 medium supplemented with 10 vol% FBS, 1 vol% NEA, 1 vol% Penicillin/Streptomycin and 1 vol% HEPES (1.00 M). HEK cells stably transduced with mCXCR4 were grown in DMEM/F12 medium with Glutamax-I, containing 10 vol% FCS and 1 vol% G418 (50 mg/mL). Cells were maintained in a humidified 5% CO_2_ atmosphere at 37°C. All media, cell culture reagents and supplements were obtained from Biochrom (Berlin, Germany) or Gibco (life technologies, Darmstadt, Germany).

**Dual tracer internalization studies**

On the day prior to the experiment, Chem-1 cells were seeded in 24-well-plates at a density of app. 1·10^5^ cells per well. On the day of the experiment, the culture medium was removed and the cells were washed once with 250 µL of unsupplemented medium before being left to equilibrate in 200 µL of assay medium (DMEM/F-12 containing 5% BSA) at 37°C for a minimum of 15 min before the experiment. Cells were then coincubated with the respective ^99m^Tc-labeled compound of interest (0.2 nM) and [^125^I]FC-131 (0.2 nM) in the absence (total binding) or presence (non-specific binding) of 100 µM AMD3100 for different time points up to 120 min at 37°C (n=3 wells, respectively, per time point). Incubation was terminated by placing the plate on an ice pack for app. 1 min and by subsequent removal of the incubation medium. Cells were thoroughly rinsed with 250 µL of HBSS. After washing twice with 250 µL of ice cold acid wash buffer (0.02 M NaOAc buffered with AcOH to pH = 5), cells were lysed with 250 µL of 1 N NaOH. The lysate was tranferred to vials and combined with 250 µL of HBSS used for rinsing the wells. Quantification of the amount of free, acid-releasable and internalized ^99m^Tc- and ^125^I-activity was performed using dual isotope protocol on a WIZARD^2®^ 2480 automatic γ-Counter (Perkin Elmer, Waltham MA, USA).

All data were corrected for non-specific internalization in the presence of 10 μM AMD3100 and were then normalized to the specific internalization observed of the standard radioligand [^125^I]FC-131.

**In vivo experiments**

**Tumor model**

To establish tumor xenografts, Jurkat cells (2 – 3x10^7^ cells) were suspended in a mixture of RPMI 1640 medium and Matrigel (1/1; v/v) (BD Biosciences, Heidelberg, Germany), and injected subcutaneously into the right shoulder of 6–10 weeks old CB17-SCID mice from either Charles River GmbH (Sulzfeld, Germany) or the in-house mouse breeding facility. As soon as tumors had grown to a diameter of 5–8 mm (4 – 10 weeks after inoculation), mice were used for the experiments.

**Patient studies**

**Whole body scintigraphy and SPECT/CT imaging protocols**

| SPECT | | 60 Views, Matrix: 128 x 128, Zoom: 1  Filter: Butterworth 0.48; Reconstruction: 2 iterations; 10 subsets | | |
| --- | --- | --- | --- | --- |
| **Time p. i.** |  | | **Scan Time** |  |
| 5 min. |  | | WB 30 cm/min. |  |
| 30 min. |  | | WB 30 cm/min. |  |
| 60 min. |  | | WB 30 cm/min. |  |
| 120 min. |  | | WB 12 cm/min. |  |
| 180 min. |  | | WB 12 cm/min.  SPECT 8 sec/View |  |
| 300 min. |  | | WB 12 cm/min. |  |
| 24 h |  | | WB 5 cm/min. |  |

**Radiation dosimetry and biodistribution**

The Xeleris^TM^ Dosimetry Toolkit (GE Healthcare, Milwaukee, USA) was used to perform full organ segmentation by an experienced user. This was performed manually on CT images of organs (kidneys, liver, spleen, heart, lungs, lumbar vertebrae 2-4) to define the regions and volumes of interest (VOIs) for gamma camera scans and SPECT analysis. Quantification of the activity in the VOIs was carried out as converting counts in the VOIs by a calibration factor to the average activity per milliliter and per organ.

The VOIs were transferred from SPECT/CT to the planar images. Additional ROIs for the whole body and a background region adjacent to the head were drawn on the planar images.

The VOIs were used to determine the masses of the individual organs. For the lungs, a density of 0.288 g/mL was used [9]. For all organs not covered by any VOI, the uptake was summed as the contribution from the remainder of the body in the dose calculation.

The time-integrated activity coefficients of all contoured organs were determined using the hybrid planar/SPECT method. Thus, the shape of the time-activity curves were estimated based on the planar images using the NUKFIT software and then scaled to activity values using the SPECT/CT. The accumulated activity in the bone marrow was calculated using the activity in the lumbar vertebrae 2-4 with the assumption that these contain 6.7% of the red bone marrow [10].

Radiation dosimetry calculations were performed using the RADAR (Radiation Dose Assessment Resource) [11-13] method as implemented in the OLINDA/EXM software and according to the recommendations of the Medical Internal Radiation Dose (MIRD) committee [11, 14, 15]. The OLINDA/EXM application (version 1.0) was used to calculate the individual absorbed organ doses (ODs) and the effective doses (EDs) by using the time-integrated activity coefficients and the individual patients’ organ masses. A mass of 1500 g for the red bone marrow as assumed for all patients. The EDs were afterwards corrected to consider the current tissue weighting factors of ICRP 103 [16]. The absorbed OD and ED were evaluated for each patient and then averaged across all the patients.

**Supplementary Tables**

***Supplementary Table 1*** *Subject details, injected [^99m^Tc]PentixaTec activity and estimated doses*

| **Patient** | **Age [y]** | **Sex** | **Weight [kg]** | **Haematocrit**  **[%]** | **Injected activity**  **[MBq]** | **Injected activity [MBq/kg BW]** | **Time since first diagnosis [months]** | **Effective dose [µSv/MBq]** | **Effective dose**  **[mSv/500 MBq]** |
| --- | --- | --- | --- | --- | --- | --- | --- | --- | --- |
| **1** | 48 | M | 102 | 34.7 | 425 | 4 | 15 | 4.26 | 2.13 |
| **2** | 69 | F | 84 | 34.9 | 567 | 7 | 35 | 6.34 | 3.17 |
| **3** | 66 | F | 97 | 26.1 | 601 | 6 | 1 | 6.70 | 3.35 |
| **4** | 72 | M | 80 | 40.0 | 417 | 5 | 14 | 4.29 | 2.15 |
| **Median** | 68 |  | 91 | 34.8 | 496 | 6 | 8 | 5.32 | 2.66 |
| **Mean** | 64 |  | 91 | 33.9 | 503 | 6 | 8 | 5.40 | 2.70 |

**Supplementary Figures
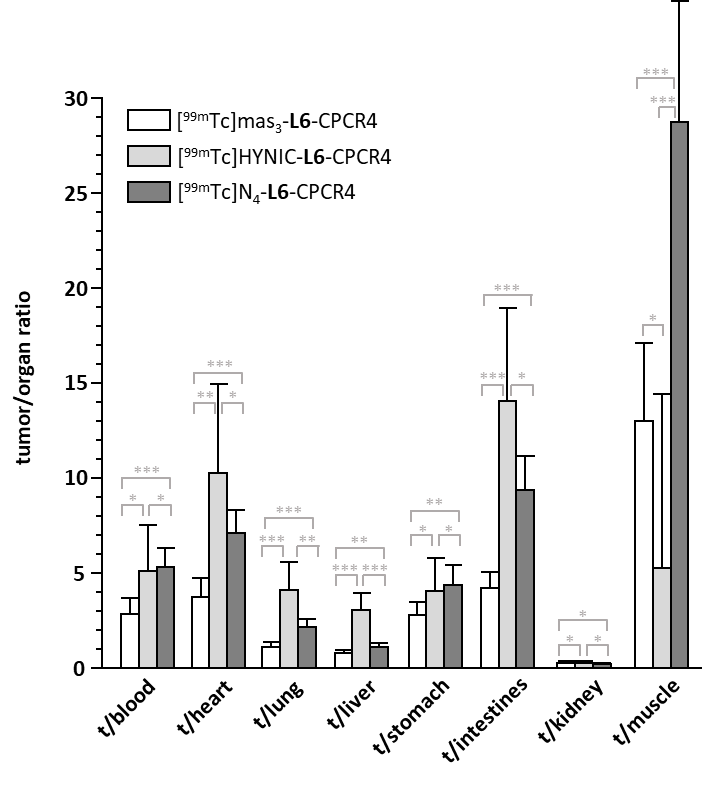
**

***Supplementary Figure 1:*** *Tumor-to-organ ratios of the different ^99m^Tc-labeled CXCR4 ligands investigated in this study. Data are means±SD (n=5 animals).*

** p > 0.05, ** 0.05 > p > 0.005, *** p < 0.005*


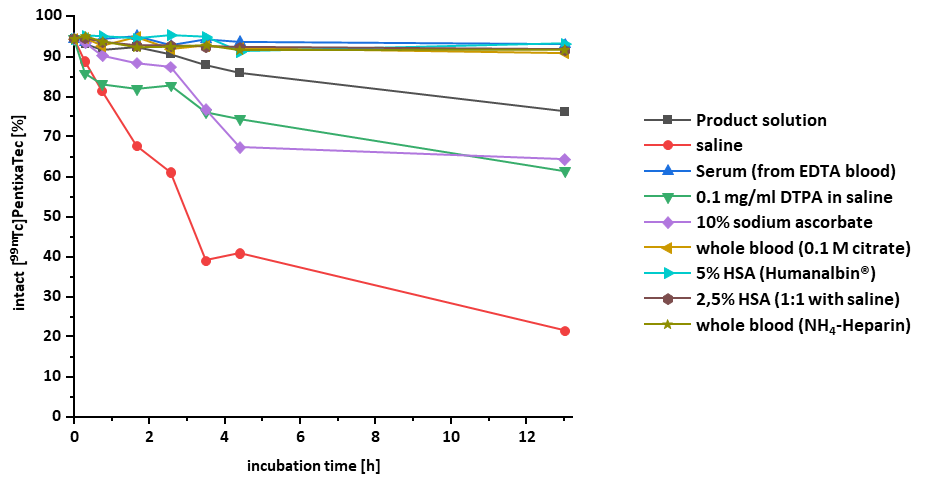


***Supplementary Figure 2:*** *in vitro stability of [^99m^Tc]PentixaTec in different physiological buffers and challenging solutions over time. Incubation was carried out at RT.*

**
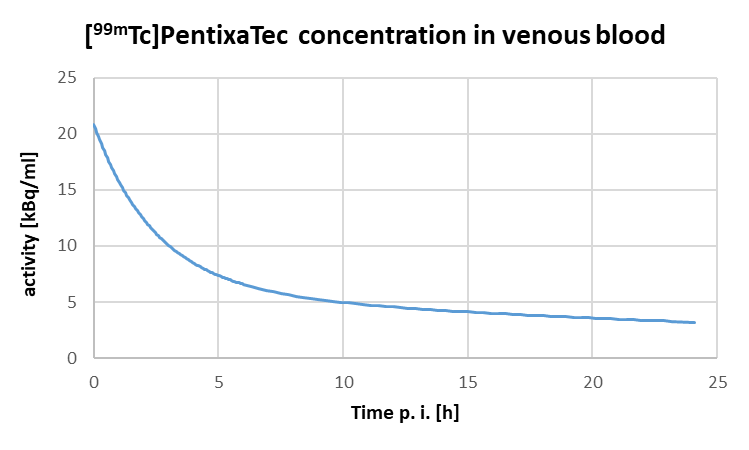
**

***Supplementary Figure 3:*** *Average activity concentration in 2.5 mL venous blood samples taken from four patients at different time points after intravenous injection of mean 503 (±82) MBg [^99m^Tc]PentixaTec.*

**References**

1. Demmer, O., Frank A.O., Hagn F., Schottelius M., Marinelli L., Cosconati S., Brack-Werner R., Kremb S., Wester H.J., Kessler H. A conformationally frozen peptoid boosts CXCR4 affinity and anti-HIV activity. *Angew Chem Int Ed Engl*. **2012**, *51*, 8110-8113.

2. Chatterjee, J., Gilon C., Hoffman A., Kessler H. N-methylation of peptides: a new perspective in medicinal chemistry. *Acc Chem Res*. **2008**, *41*, 1331-1342.

3. Abrams, M.J., Juweid M., tenKate C.I., Schwartz D.A., Hauser M.M., Gaul F.E., Fuccello A.J., Rubin R.H., Strauss H.W., Fischman A.J. Technetium-99m-human polyclonal IgG radiolabeled via the hydrazino nicotinamide derivative for imaging focal sites of infection in rats. *Journal of Nuclear Medicine*. **1990**, *31*, 2022-2028.

4. Joyard, Y., Bischoff L., Levacher V., Papamicel C., Vera P., Bohn P. Synthesis and Stability Evaluation of New HYNIC Derivatives as Ligands for Technetium-99m. *Letters in Organic Chemistry*. **2014**, *11*, 208-214.

5. Abiraj, K., Mansi R., Tamma M.L., Forrer F., Cescato R., Reubi J.C., Akyel K.G., Maecke H.R. Tetraamine-derived bifunctional chelators for technetium-99m labelling: synthesis, bioconjugation and evaluation as targeted SPECT imaging probes for GRP-receptor-positive tumours. *Chemistry*. **2010**, *16*, 2115-2124.

6. Schottelius, M., Ludescher M., Richter F., Kapp T.G., Kessler H., Wester H.J. Validation of [(125)I]CPCR4.3 as an investigative tool for the sensitive and specific detection of hCXCR4 and mCXCR4 expression in vitro and in vivo. *EJNMMI Res*. **2019**, *9*, 75.

7. Robu, S., Schottelius M., Eiber M., Maurer T., Gschwend J., Schwaiger M., Wester H.J. Preclinical Evaluation and First Patient Application of 99mTc-PSMA-I&S for SPECT Imaging and Radioguided Surgery in Prostate Cancer. *Journal of Nuclear Medicine*. **2017**, *58*, 235-242.

8. Kuzmanovska, S., Vaskova O., Zdraveska Kocovska M. “In-house” preparation of 99mTc-EDDA/HYNIC-TOC, a specific targeting agent for somatostatin receptor scintigraphy. *Macedonian Pharmaceutical Bulletin* **2011**, *57*, 65-70.

9. Guenard, H., Diallo M.H., Laurent F., Vergeret J. Lung density and lung mass in emphysema. *Chest*. **1992**, *102*, 198-203.

10. Herrmann, K., Lapa C., Wester H.J., Schottelius M., Schiepers C., Eberlein U., Bluemel C., Keller U., Knop S., Kropf S., Schirbel A., Buck A.K., Lassmann M. Biodistribution and radiation dosimetry for the chemokine receptor CXCR4-targeting probe 68Ga-pentixafor. *Journal of Nuclear Medicine*. **2015**, *56*, 410-416.

11. Siegel, J.A., Thomas S.R., Stubbs J.B., Stabin M.G., Hays M.T., Koral K.F., Robertson J.S., Howell R.W., Wessels B.W., Fisher D.R., Weber D.A., Brill A.B. MIRD pamphlet no. 16: Techniques for quantitative radiopharmaceutical biodistribution data acquisition and analysis for use in human radiation dose estimates. *Journal of Nuclear Medicine*. **1999**, *40*, 37S-61S.

12. Stabin, M.G., Sharkey R.M., Siegel J.A. RADAR commentary: Evolution and current status of dosimetry in nuclear medicine. *Journal of Nuclear Medicine*. **2011**, *52*, 1156-1161.

13. Stabin, M.G., Xu X.G., Emmons M.A., Segars W.P., Shi C., Fernald M.J. RADAR reference adult, pediatric, and pregnant female phantom series for internal and external dosimetry. *Journal of Nuclear Medicine*. **2012**, *53*, 1807-1813.

14. Stabin, M.G. Uncertainties in internal dose calculations for radiopharmaceuticals. *Journal of Nuclear Medicine*. **2008**, *49*, 853-860.

15. Loevinger, R., Budinger T.F., Watson E.E., Committee S.o.N.M.M.I.R.D. MIRD Primer for Absorbed Dose Calculations. Society of Nuclear Medicine, 1991.

16. The 2007 Recommendations of the International Commission on Radiological Protection. ICRP publication 103. *Ann ICRP*. **2007**, *37*, 1-332.
